# Aspermerodione, a novel fungal metabolite with an unusual 2,6-dioxabicyclo[2.2.1]heptane skeleton, as an inhibitor of penicillin-binding protein 2a

**DOI:** 10.1038/s41598-018-23817-1

**Published:** 2018-04-03

**Authors:** Yuben Qiao, Xiaotian Zhang, Yan He, Weiguang Sun, Wenya Feng, Junjun Liu, Zhengxi Hu, Qianqian Xu, Hucheng Zhu, Jinwen Zhang, Zengwei Luo, Jianping Wang, Yongbo Xue, Yonghui Zhang

**Affiliations:** 1 Hubei Key Laboratory of Natural Medicinal Chemistry and Resource Evaluation, School of Pharmacy, Tongji Medical College, Huazhong University of Science and Technology, Wuhan, 430030 Hubei Province People’s Republic of China; 2 Biological Engineering, Wuchang Shouyi University, Wuhan, 430064 China; 3 Tongji Hospital Affiliated to Tongji Medical College, Huazhong University of Science and Technology, Wuhan, 430030 People’s Republic of China

## Abstract

Rising drug resistance limits the treatment options infected by methicillin-resistant *Staphylococcus aureus* (MRSA). A promising solution for overcoming the resistance of MRSA is to inhibit the penicillin-binding protein 2a (PBP2a). A novel terpene-polyketide hybrid meroterpenoid, aspermerodione (**1**), characterized by an unusual 2,6-dioxabicyclo[2.2.1]heptane core skeleton, and a new heptacyclic analogue, andiconin C (**2**), were isolated and identified from the liquid cultures of endophytic fungus *Aspergillus* sp. TJ23. The structures and their absolute configurations of all chiral centers were elucidated via extensive spectroscopic analyses and electronic circular dichroism (ECD) calculations and determined via single-crystal X-ray diffraction analysis. Aspemerodione (**1**) was found to be a potential inhibitor of PBP2a, and work synergistically with the *β*-lactam antibiotics oxacillin and piperacillin against MRSA.

## Introduction

Methicillin-resistant *Staphylococcus aureus* (MRSA) is one of the most prevalent multidrug-resistant pathogens worldwide, and it represents a major public health problem^1^. Over the past several decades, the emergence of methicillin-resistant MRSA and its rapid spread has led to the increasing difficulty in treating MRSA infection^2,3^. MRSA acquires a series of drug-resistance gene mutations, as exemplified by *mec*A, and encodes penicillin-binding protein 2a (PBP2a), which is resistant to most *β*-lactam antibiotics^4^. PBP2a specifically plays vital role on crosslinking of the cell wall, even when other PBPs are inhibited by *β*-lactam antibiotics^5,6^. The underlying mechanism is involved in allostery mediated inactivated conformation for the active site^7,8^.

Recent studies have demonstrated that the combined use of small molecules that bind to the allosteric active site of PBP2a can render MRSA sensitive to the effects of conventional *β*-lactam antibiotics^9,10^. To this end, the inhibition of PBP2a is a hopeful solution for overcoming the multiple drug resistance of MRSA via preserving the availability of therapeutic agents that are already useful in the pharmacological armamentarium^9^.

In our efforts to identify novel and bioactive compounds from Chinese traditional medicins (TCMs) and their endophytic microbial sources^11–13^, an extensive biological screen against MRSA (ATCC43300) *in vitro* was conducted, and the endophytic fungus *Aspergillus* sp. TJ23 showed the best bioactivity against the indicator bacteria. Spiroaspertrione A, a metabolite of *Aspergillus* sp. TJ23 that has a novel terpene–polyketide skeleton, shows potent resensitisation of oxacillin against MRSA^13^. Since the secondary metabolites was significantly effected by the culture medium^14^, we preliminarily investigated the influence on the secondary productions of the endophytic fungus *Aspergillus* sp. TJ23 in a liquid broth.

**Figure 1:**
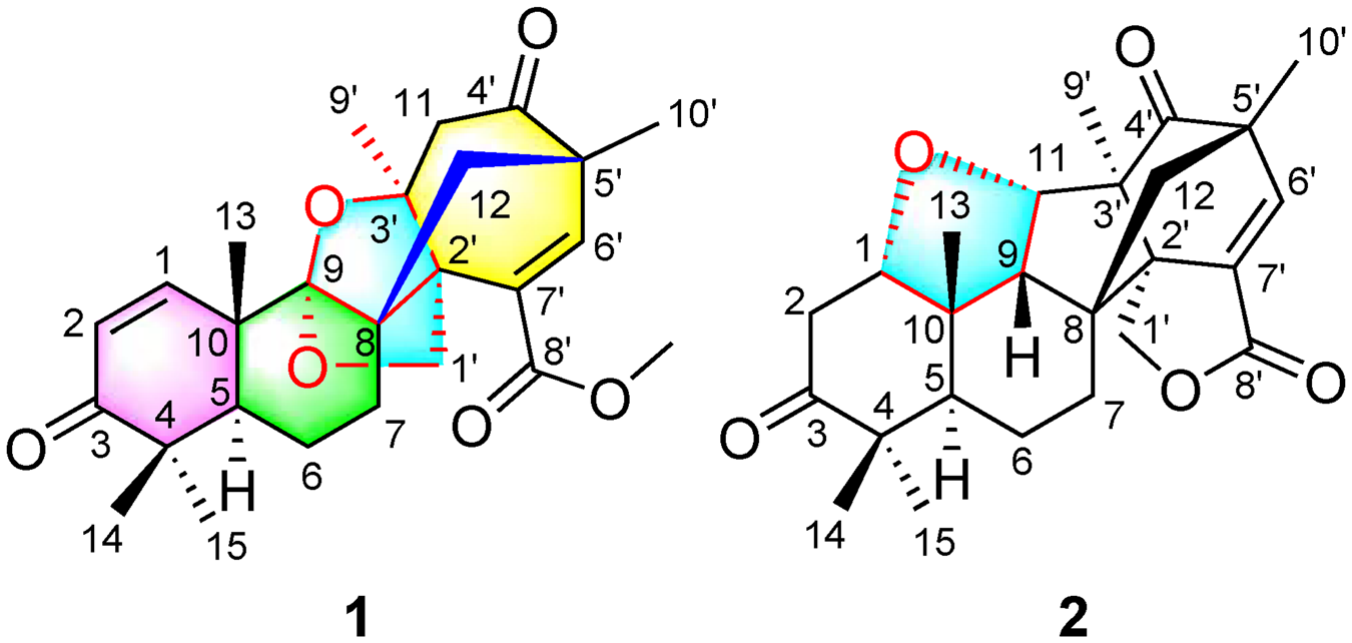
Structures of compounds **1** and **2**.

Isolation of the liquid cultures of this fungal strain yielded two polycyclic terpene–polyketide hybrid meroterpenoids (Fig. 1), aspermerodione (**1**), which is characterized by the incorporation of a rare 2,6-dioxabicyclo[2.2.1]heptane group into the spiro[bicyclo[3.2.2]nonane-2,1′-cyclohexane] ring system, and a new heptacyclic analogue, andiconin C (**2**). Herein, the isolation, structural elucidation, plausible biosynthetic pathway, and bioactivities of compounds **1** and **2** are presented.

## Results and Discussion

### Structural elucidation of the isolates

Compound **1** was isolated as colorless crystals (in CHCl_3_–MeOH). The molecular formula of **1** was determined to be C_26_H_32_O_6_ by the positive ion peaks at *m/z* 441.2251 [M + H]^+^ and 463.2049 [M + Na]^+^ in the HRESIMS spectrum, denoting 11 degrees of unsaturation. The ^1^H NMR spectrum (Table 1) of **1** demonstrated six sp^3^ singlet methyls at *δ*_H_ 1.07 (H_3_-14), 1.12 (H_3_-15), 1.21 (H_3_-13), 1.29 (H_3_-10′), 1.45 (H_3_-9′), and 3.73 (OCH_3_-8′), and three olefinic protons at *δ*_H_ 7.27 (d, *J* = 10.0, H-1), 5.87 (d, *J* = 10.0, H-2), and 6.87 (s, H-6′). Additionally, the ^13^C NMR and DEPT spectra (Table 1) revealed 26 carbon atom resonances corresponding to six sp^3^ methyls (including one oxygenated), five methylenes (including one oxygenated), one sp^3^ methine at *δ*_C_ 46.5 (C-5), and seven sp^3^ quaternary carbons [including one ketal carbon at *δ*_C_ 109.3 (C-9)]. Three carbonyl groups and four olefinic carbon atoms, accounting for 5 out of 11 degrees of unsaturation, indicated that **1** possessed a hexacyclic ring system.

**Table 1:** ^1^H NMR (400 MHz) and ^13^C NMR (100 MHz) spectroscopic data for compounds **1** and **2**.

No.	**1** (in CDCl_3_)		**2** (in CDCl_3_)	
	*δ*_H_ *J* in Hz	*δ*_C_ type	*δ*_H_ *J* in Hz	*δ*_C_ type
1	7.27, d (10.0)	155.4, CH	3.62, t (7.1)	84.1, CH
2	5.87, d (10.0)	126.2, CH	2.76, overlap; 2.78, overlap	40.0, CH_2_
3		204.2, C		214.4, C
4		44.3, C		46.0, C
5	2.33, m	46.5, CH	1.78, m	41.6, CH
6	1.67, m	18.5, CH_2_	1.60, m; 1.71, m	17.5, CH_2_
7	1.25, m; 1.62, m	30.9, CH_2_	1.78, m; 1.93, m	29.3, CH_2_
8		53.5, C		57.1, C
9		109.3, C	1.96, d (6.6)	65.1, CH
10		41.6, C		45.5, C
11	2.50, d (19.0)	51.2, CH_2_	3.95, d (6.6)	86.9, CH
	2.65, d (19.0)			
12	1.46, overlap	38.1, CH_2_	1.56, d (5.2)	59.2, CH_2_
	2.20, d (14.9)		1.61, d (5.2)	
13	1.21, s	18.9, CH_3_	0.90, s	20.9, CH_3_
14	1.07, s	21.2, CH_3_	1.01, s	18.5, CH_3_
15	1.12, s	28.3, CH_3_	1.14, s	28.5, CH_3_
1′	4.03, d (9.1)	67.2, CH_2_	4.71, d (10.4)	70.3, CH_2_
	4.69, d (9.1)		5.93, d (10.4)	
2′		55.8, C		59.7, C
3′		84.1, C		46.1, C
4′		200.6, C		211.2, C
5′		50.8, C		52.2, C
6′	6.87, s	145.3, CH	7.08, s	142.7, CH
7′		130.3, C		134.3, C
8′		164.6, C		166.9, C
9′	1.45, s	27.6, CH_3_	1.18, s	13.6, CH_3_
10′	1.29, s	22.3, CH_3_	1.31, s	16.9, CH_3_
OCH_3_-8′	3.73, s	52.0, CH_3_		

**Figure 2:**
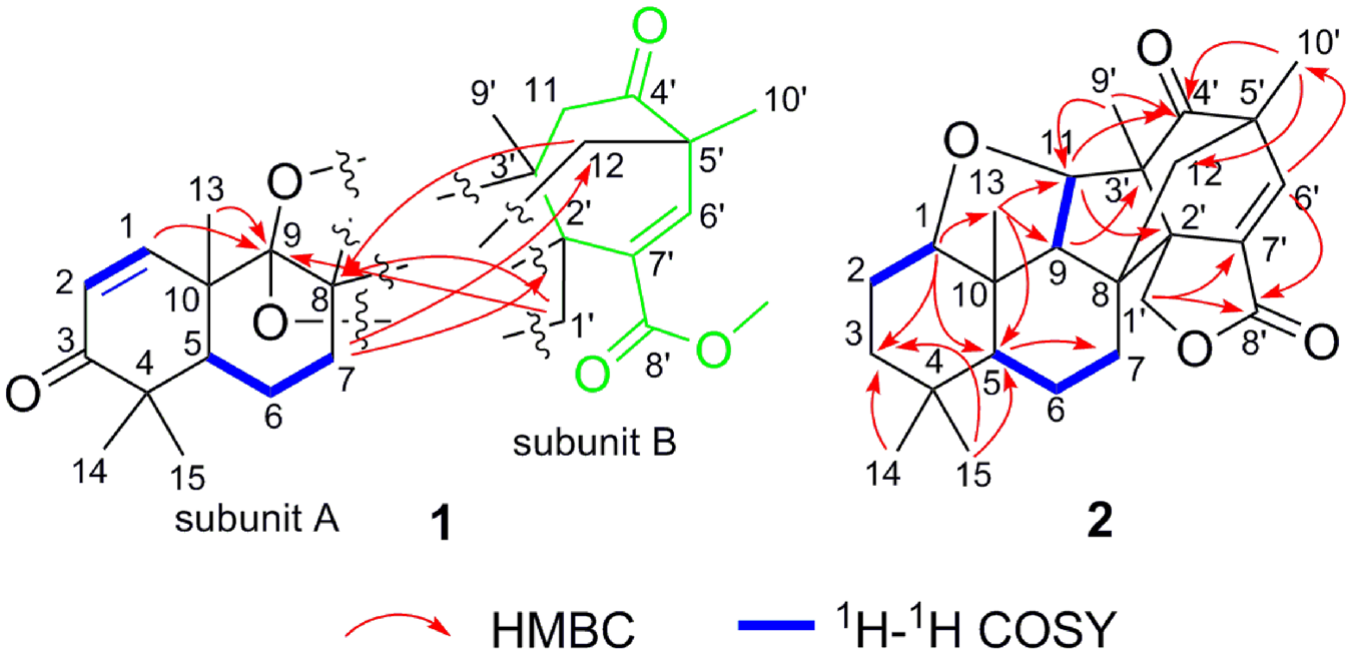
Key HMBC and ^1^H**–**^1^H COSY correlations of compounds **1** and **2**.

All proton resonances were assigned to their respective carbons with the help of HSQC spectrum. Comparison of the HRESIMS and spectroscopic data of **1** with previously reported meroterpenoids^15^, prompted us to consider that compound **1** was an unusual rearranged meroterpenoid comprising two subunits, A and B, which were subsequently ascertained as follows (Fig. 2 and Fig. S19, Supplementary Information). The key HMBC correlations from H-1, H_3_-14, and H_3_-15 to C-3 and C-5; from H-2, H_3_-14, and H_3_-15 to C-4; from H-5 to C-7, C-10, and C-13; and from H_3_-13 to C-1, C-5, C-9, and C-10, combined with the spin-spin coupling systems of H-1/H-2 and H-5/H_2_-6/H_2_-7 in its ^1^H–^1^H COSY spectrum, indicated the presence of subunit A. In addition, the subunit B was constructed by the key HMBC correlations from H_3_-9′ to C-2′, C-3′, and C-11; from H_3_-10′ to C-4′, C-6′, and C-12; from H-6′ to C-2′, C-4′, C-8′, and C-10′; from H_2_-12 to C-4′ and C-6′, and from OCH_3_-8′ to C-8′. The fusion of subunits A and B was deduced based on the obvious HMBC (Fig. 2) correlations from H_2_-12 and H_2_-1′ to C-8 and C-9, which displayed the direct carbon-carbon connectivity of C-12 and C-8 and ether bridge linkage between C-1′ and C-9. Given the index of hydrogen deficiency of 11 from its molecular formula, the carbon resonance of C-9 (*δ*_C_ 109.3) in **1** was observed to significantly shift downfield, to which we assigned the existence of another *O*-linkage at C-9/C-3′ to generate an unusual 2,6-dioxabicyclo[2.2.1]heptane core scaffold. Thus, the planar structure of **1** was established as shown in Fig. 1.

**Figure 3:**
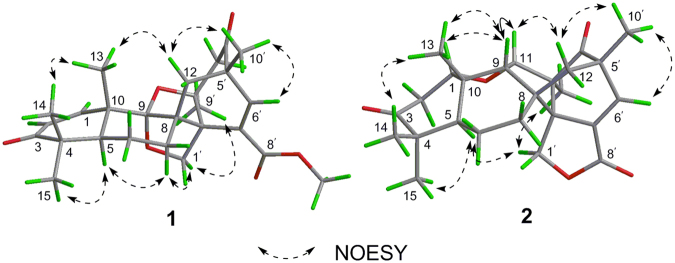
Key NOESY correlations of compounds **1** and **2**.

Based on a detailed analysis of the NOESY (Fig. 3) spectrum of **1** and referring the bio-origin of paralleled meroterpenoids^13,15^, the relative configuration of **1** was established. Structurally, the relative configuration of H-5 of paralleled meroterpenoids was *α*-oriented^15,16^. The obvious NOESY correlations (Fig. 3) of H-5/H_3_-15, H-5/H_2_-7, H_2_-7/H_2_-1′, and of H_2_-1′/H_3_-9′, indicated that these protons are all on the same side of molecule and assigned as the *α*-orientation. In contrast, the key NOESY correlations from H_3_-13 to H_3_-14 and H_2_-12 displayed in its NOESY spectrum (Fig. 3), showed they are on the opposite face on the molecule and assigned to the *β*-orientation. The relative configuration of **1** was thus established as 2′R^*^,3′S^*^,5S^*^,5′S^*^,8R^*^,9R^*^,10S^*^.

**Figure 4:**
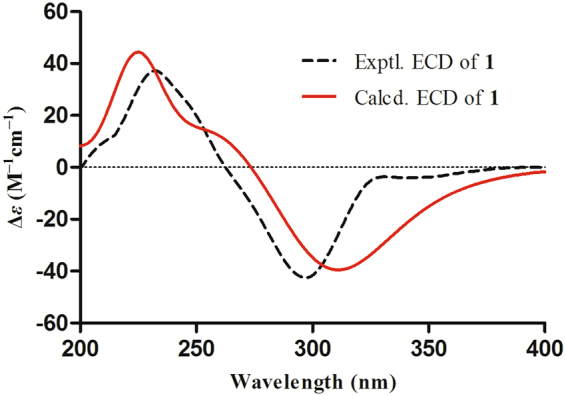
Experimental and calculated ECD spectra of **1** recorded in MeOH.

To elucidate the absolute configurations of all chiral centers of **1**, a time-dependent density functional theory (TDDFT) method at the B3LYP/6-311++G** level with PCM in MeOH was performed for 2′*R*,3′*S*,5 *S*,5′*S*,8 *R*,9 *R*,10*S*-**1** (Fig. 4), in which the experimental ECD spectrum of **1** was in good agreement with its calculated ECD curve, indicating that the absolute configuration of **1** was 2′*R*,3′*S*,5 *S*,5′*S*,8 *R*,9 *R*,10 *S* (Fig. 1).

**Figure 5:**
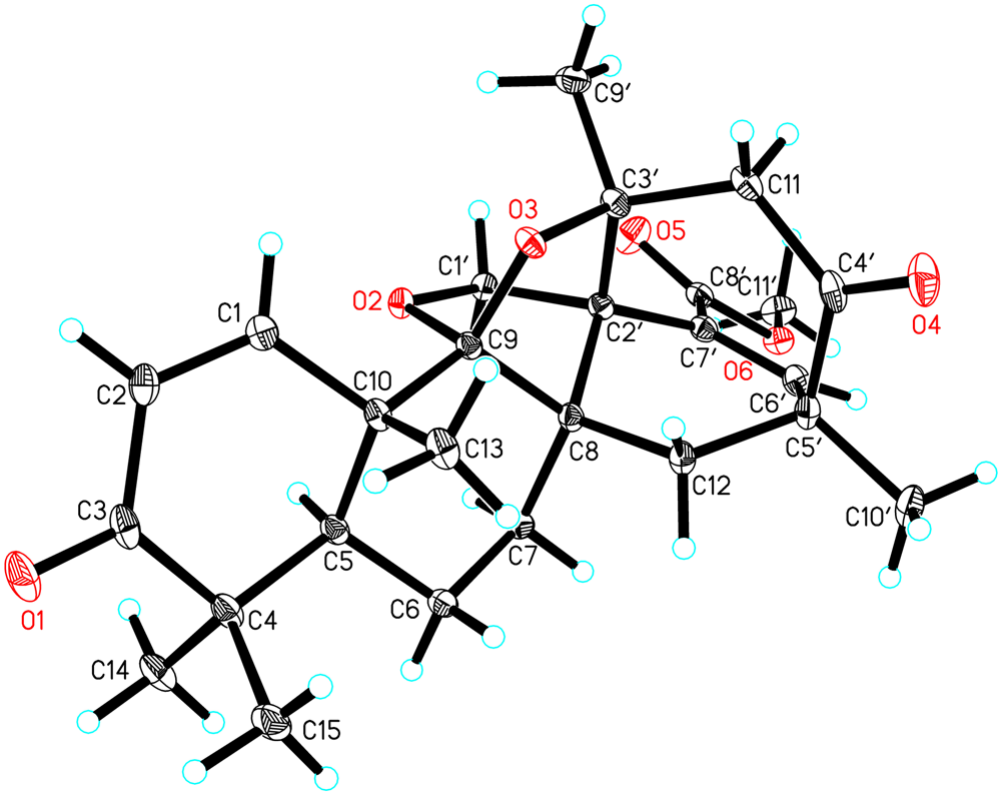
X-ray ORTEP drawing of compound **1**.

A suitable crystal of **1** was obtained by slow evaporation of a mixture of CHCl_3_–MeOH (10:1) at room temperature, and subjected to a single-crystal X-ray diffraction experiment using Cu K*α* radiation (Fig. 5, CCDC 1554255)^17^, which supported the proposed structure and its absolute configuration. It was given the name aspermerodione, which featured the incorporation of a rare 2,6-dioxabicyclo[2.2.1]heptane moiety into the spiro[bicyclo[3.2.2]nonane-2,1′-cyclohexane] ring system.

Compound **2** was obtained as an optically active, colorless crystal (in CHCl_3_–MeOH). The HRESIMS analysis of **2** displayed a sodiated ion peak at *m/z* 433.1961 [M + Na]^+^ and a protonated ion peak at *m/z* 411.2146 [M + H]^+^, and we determined its molecular formula to be C_25_H_30_O_5_, denoting 11 degrees of unsaturation. The ^1^H NMR spectrum (Table 1) of **2** showed signals attributed to five singlet methyls at *δ*_H_ 0.90 (H_3_-13), 1.01 (H_3_-14), 1.14 (H_3_-15), 1.18 (H_3_-9′), and 1.31 (H_3_-10′), one oxygenated sp^3^ methylene at *δ*_H_ 4.93 and 4.71 (both d, *J* = 10.4 Hz, H_2_-1′), two oxygenated sp^3^ methines at *δ*_H_ 3.62 (t, *J* = 7.1 Hz, H-1) and *δ*_H_ 3.95 (d, *J* = 6.5 Hz, H-11), and one olefinic proton signals at *δ*_H_ 7.08 (s, H-6′). The ^13^C NMR and DEPT spectra of **2** (Table 1), displayed resonances for 25 carbon signals that were resolved into three carbonyl carbons at *δ*_C_ 166.9 (C-6′), 211.2 (C-4′), and 214.4 (C-3), five methyl groups at *δ*_C_ 13.6 (C-9′), 16.9 (C-10′), 18.5 (C-14), 20.9 (C-13), and 28.5 (C-15), five methylenes including one oxygenated [*δ*_C_ 17.5 (C-6), 29.3 (C-7), 40.0 (C-2), 59.2 (C-12), and 70.3 (C-1′)], two olefinic carbon signals at *δ*_C_ 142.7 (C-6′) and 134.3 (C-7′), four methines including two oxygenated [*δ*_C_ 41.6 (C-5), 65.1 (C-9), 84.1 (C-1), and 86.9 (C-1)], and six sp^3^ quaternary carbon atoms at *δ*_C_ 45.5 (C-10), 46.0 (C-4), 46.1 (C-3′), 52.2 (C-5′), 57.1 (C-8), and 59.7 (C-2′). The aforementioned spectroscopic data of **2** showed signals similar to those of the known compound, andiconin B^13^, indicating that both share an andiconin-type carbocyclic skeleton, except that a couple of double peak olefinic proton signals (both d, *J* = 10.3 Hz) and a sp^3^ methine group in andiconin B were replaced by an oxygenated methine and a singlet olefinic proton in **2**. Those variations and one additional degree of unsaturation when compared to andiconin B suggested an oxygen linkage between C-1 and C-11 to form a new ring system and demonstrated the formation of a new double bond with dehydration between C-6′ and C-7′, which was evidenced by the relative downfield shifted carbon resonances of C-6′, C-7′, and C-11, and supported by the significant up-field chemical shifts from 152.6 and 126.5 ppm to 84.1 and 40.0 ppm, respectively, in **2**. This deduction was further verified by the key HMBC (Fig. 2 and Fig. S19, Supplementary Information) correlations from H-1 to C-3, C-5. C-9, C-10, and C-13, and from H-6′ to C-2′, C-4′, C-5′, C-8′, C-10′, and C-12, and further supported by the spin-spin coupling systems of H-1/H_2_-2 and H-9/H-11 in its ^1^H–^1^H COSY spectrum (Fig. 2). Thus, the planar structure of **2** was elucidated as shown (Fig. 1).

The relative configuration of **2** was determined by the comprehension of the key NOESY spectrum (Fig. 3) and coupling constants. The obvious NOEs of H-5 with H_3_-15 and H_2_-7, H_2_-7 with H_2_-1′, and H_2_-1′ with H_3_-9′ indicated that those protons were all arbitrarily assigned to the *α*-orientation. Accordingly, the NOESY correlations of H-1/H_3_-14/H_3_-13, H-11/H_2_-12, and of H-9 with H_3_-13 and H-11 suggested that they were in the *β* configuration.

**Figure 6:**
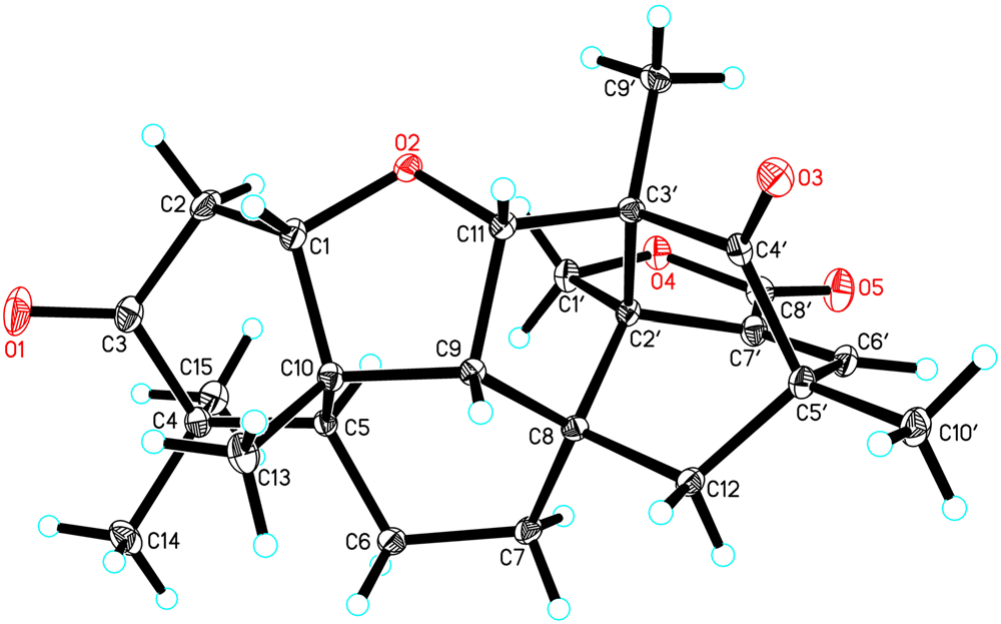
X-ray ORTEP drawing of compound **2**.

**Figure 7:**
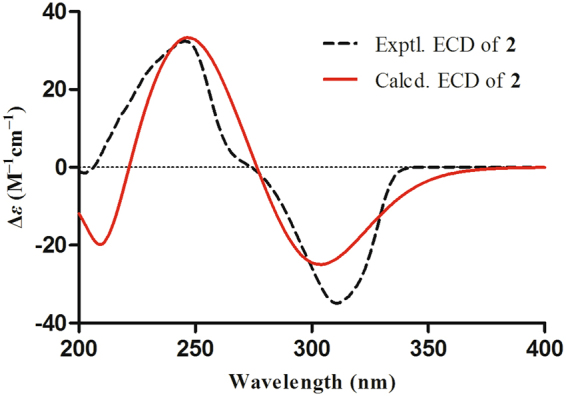
Experimental and calculated ECD spectra of **2** recorded in MeOH.

After repeated recrystallization by various solvent systems, **2** furnished a high-quality crystal in a mixture of CHCl_3_–MeOH (7:3) at room temperature, which was successfully subjected to single-crystal X-ray diffraction using Cu K*α* radiation (Fig. 6) with a Flack parameter of –0.03(5) (CCDC 1554256)^17^, which enabled us to confirm its absolute configuration as 1*S*,2′*R*,3′*S*,5*R*,5′*S*,8*S*,9*R*,10*S*,11*S* (Fig. 1). To further support this deduction, the calculated ECD spectrum of 1*S*,2′*R*,3′*S*,5*R*,5′*S*,8*S*,9*R*,10*S*-**2** was determined at the B3LYP/6–31 G(d,p) level and showed an excellent coincidence with the experimental ECD curve (Fig. 7), which undoubtedly corroborated its absolute structure.

**Figure 8:**
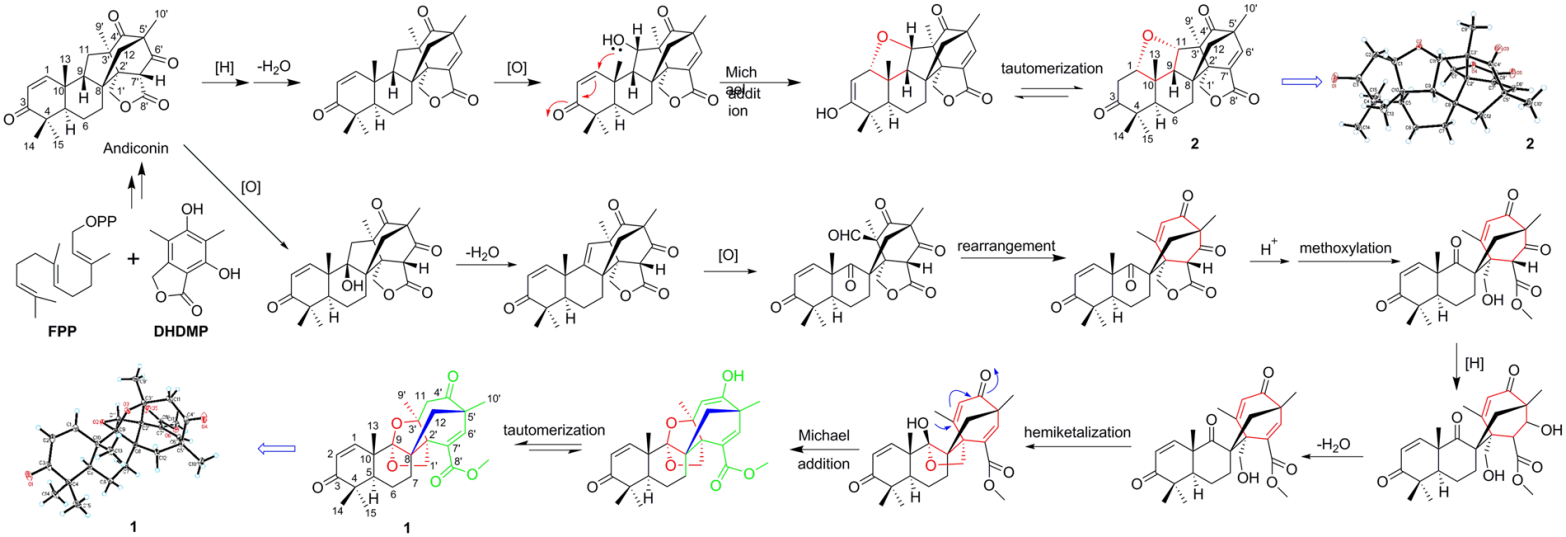
Plausible biosynthetic pathway for compounds **1** and **2**.

To our knowledge, aspermerodione (**1**) represents a novel meroterpenoid with an unusual 2,6-dioxabicyclo[2.2.1]heptane carbocyclic core. The biosynthetic origin for **1** was proposed with the co-isolated **2** as a bio-analogue (Fig. 8). Derived from farnesyl pyrophosphate (FPP) and HDMP^15,16^, a series of alkylation and intramolecular cyclization reactions led to the formation of the crucial biosynthetic intermediate, andiconin. Andiconin subsequently participated in many steps of reactions involving oxidation, methoxylation, hemiketal, and Michael addition reactions to furnish the polycyclic scaffold of **1**. In addition, andiconin could also be transformed to **2** via a series of reactions including reduction, dehydration, oxidation, Michael addition, and keto-enol tautomerism.

### Antimicrobial activity

Antibacterial activities against MRSA were observed for **1** with MIC values of 32 *μ*g/ml, whereas compound **2** showed only marginal antimicrobial activity. In addition, compound **1** was found to work synergistically with the *β*-lactam antibiotics oxacillin and piperacillin with ∑FIC values that were less than 0.5 (Table 2)^14^. To further investigate the antibacterial mechanism of **1**, *in silico* target confirmation assays^13^ were employed to forecast its possible targets. Therefore, **1** was screened against all five PBPs (PBP1, PBP2, PBP2a, PBP3, and PBP4) encoded in the MRSA core genome *in silico*^8^, which were considered to be important or promising targets for staphylococcal *β*-lactam resistance^9,10,18,19^.

**Table 2:** Combination susceptibility test of compounds **1** and **2** with various antibacterial antibiotics against MRSA in Checkerboard assay.

Antibiotics		MIC (*μ*g/mL)^a^		∑FIC_1+A_^c^	∑FIC_2+A_^c^
	Antibiotic alone (A)	A + Cmpd **1**	A + Cmpd **2**		
Oxacillin	32	4^b^	16	0.25*	>0.5
Piperacillin	64	4	32	0.375*	>0.5
Chloramphenicol	16	4	16	1.25	≥2.0
Vancomycin	0.5	0.5	0.5	>1.0	>1.0

^a^The MIC of compounds **1** and **2** alone against MRSA were 32 *μ*g/mL and >100 *μ*g/mL, respectively.

The MIC of oxacillin combined with compound **1** against MRSA was 1 *μ*g/mL (oxacillin and **1** were co-dosed at sub-MIC levels, both 1 *μ*g/mL).

^*^Compound **1** was determined to work synergistically with oxacillin and piperacillin, with ∑FIC values of 0.25 and 0.375 However, it did not show any synergistic effect on other antibiotics.

^c^∑FIC = FIC_Cmpd_ + FIC_Antibiotic_; FIC_Cmpd_ = [MIC_Cmpd_ in combination]/[MIC_Cmpd_ alone]; FIC_Antibiotic_ = [MIC_Antibiotic_ in combination]/[MIC_Antibiotic_ alone]. The combination is considered synergistic when the ∑FIC value is ≤0.5, accumulative or indifferent when the ∑FIC value is >0.5 but <2, and antagonistic when the ∑FIC is ≥2.

### Inverse docking identified the possible antibacterial target

**Figure 9:**
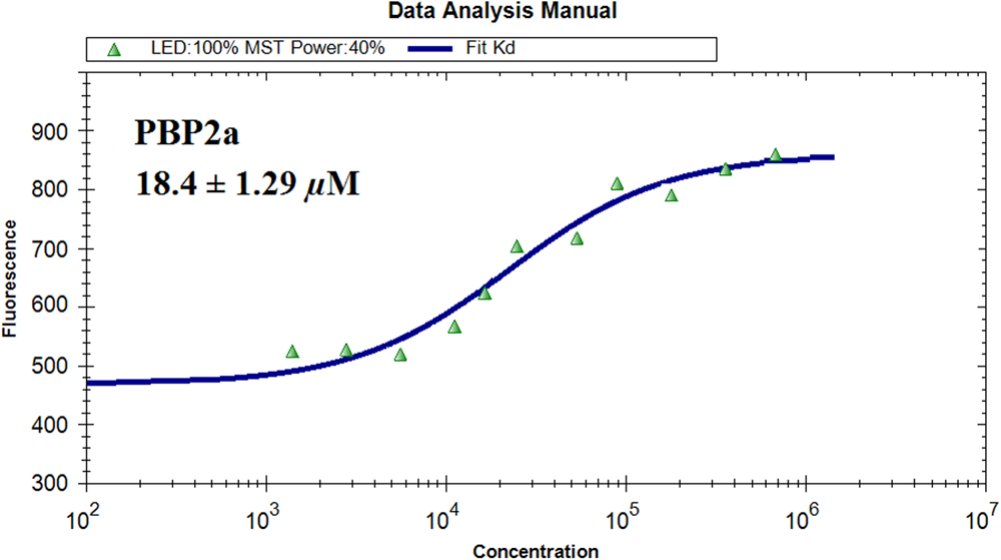
MST confirmed that compound **1** maintained specific binding to MRSA PBP2a. The measurement of the affinity between compound **1** with PBP2a via MST in standard treated capillaries is shown in the resulting binding curve. From the resulting binding curve, Kd = 18.4 ± 1.29 *µ*M for compound **1**.

The potent antimicrobial activity of compound **1** prompted us to investigate the underlying mechanism. As mentioned above, some complexes of PBPs (PDB ID: 5TRO, 2ZC3, 4CJN, 3PBR and 3HUM) from *S*. *aureus* were applied in virtual screening. The total-score values (Table 3) predicted that compound **1** was most likely to bind to allosteric site of PBP2a with higher total score value. To further define this speculation, a microscale thermophoresis (MST) method, which has previously been used to investigate protein-protein, small organic molecule-protein and antibody-protein interactions, was employed to assay the binding affinity between the compounds and PBPs^12^. As a result, **1** exhibited the strongest binding with PBP2a (Table 3). The equilibrium dissociation constant (Kd) of **1** was 18.4 ± 1.29 *μ*M (Fig. 9).

**Table 3:** Predicted binding free energies of compound **1** with five PBPs of MRSA, which considered to be important or possible media for staphylococcal *β*-lactam resistance. (Surflx-Dock scores)^a^.

PDB ID	Protein name	Compound 1
5TRO	PBP1	2.431
2ZC3	PBP2	1.327
4CJN	PBP2^a^	5.163*
3PBR	PBP3	4.129
3HUM	PBP4	2.017

^a^Docking score/interaction potential of compounds with targets (kcal/mol).

^*^The docking scores predicted that PBP2a showed significantly higher binding affinity for **1** than other PBPs.

**Figure 10:**
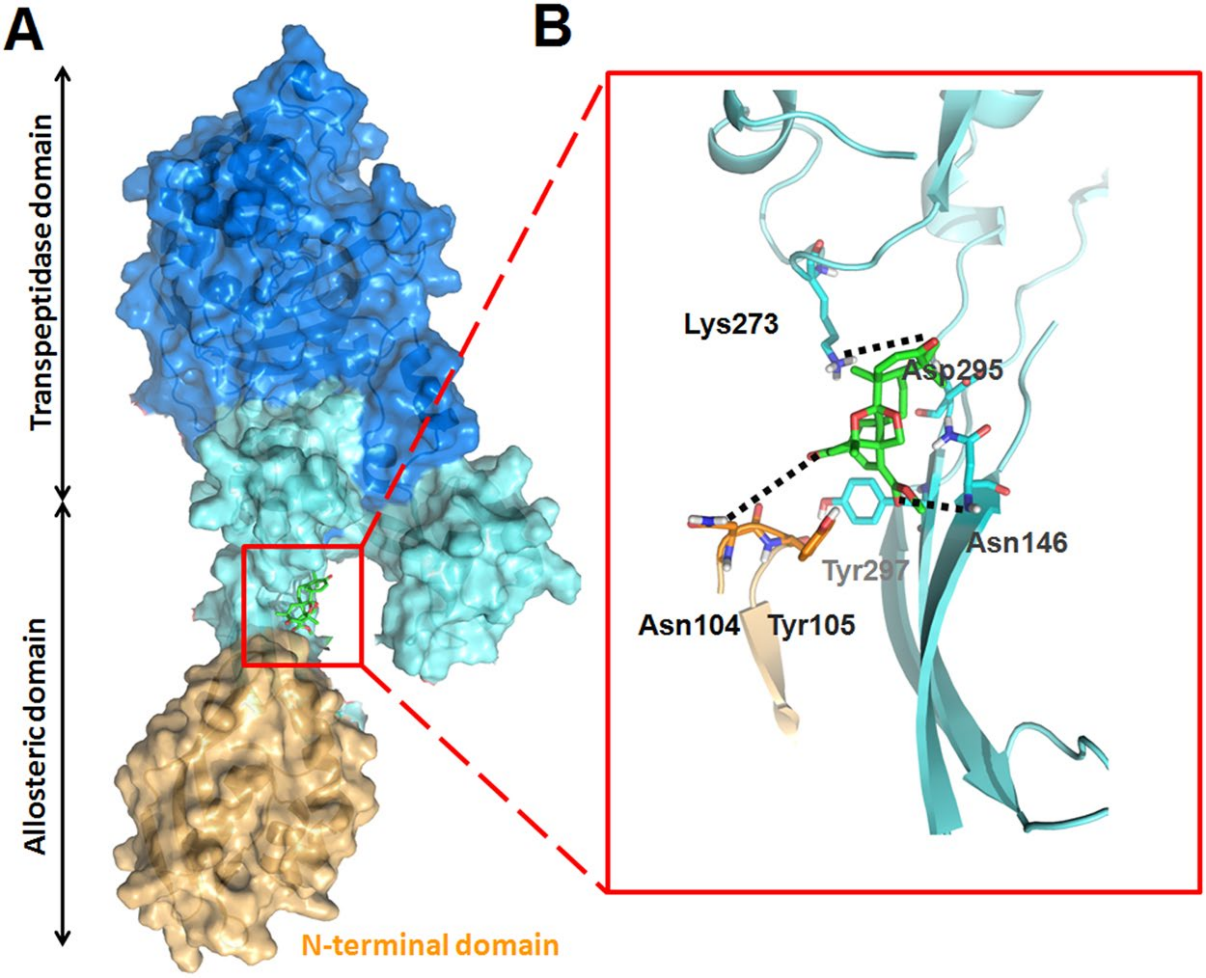
Docking model of PBP2a in complex with compound **1**. (**A**) Ribbon representation of PBP2a showing compound **1** (in green for carbons) bound to the allosteric site. The allosteric domain spans residues 27−326, where the N-terminal domain (residues 27−138) is shown in yellow and the remaining allosteric domain is light blue colored. The transpeptidase domain (residues 327−668) is shown in dark blue. (**C**) Key interactions of compound **1** at the allosteric site. Hydrogen-bond interactions with Asn104, Asn146 and Lys273 are shown as black dashed lines.

Previous research showed the PBP2a as an elongated protein with a transpeptidase domain (residues 327–668) and a nonpenicillin-binding domain (residues 27–326), which includes an N-terminal extension subdomain (residues 27–138). Two major binding sites of PBP2a had been identified: one at the active site, where binding of antibiotic or peptidoglycan is known to take place, and another within the nonpenicillin-binding domain, 60 Å distant from the active site, which was proved to be allosteric site. The further virtual docking revealed that compound **1** possibly bound to the allosteric site of PBP2a, further lead to the opening of the active site, enabling catalysis by PBP2a. This result was consistent with synergistic effect of compound **1** with *β*-lactam antibiotics. As shown in Fig. 10, key hydrogen-bond interactions were between compound **1** with the residues of Asn104, Asn146 and Lys273. Based on the molecular docking results, importing hydrophilic group substituents placed at the C-6 and C-7 position is a worthy target for further structural modification.

**Figure 11:**
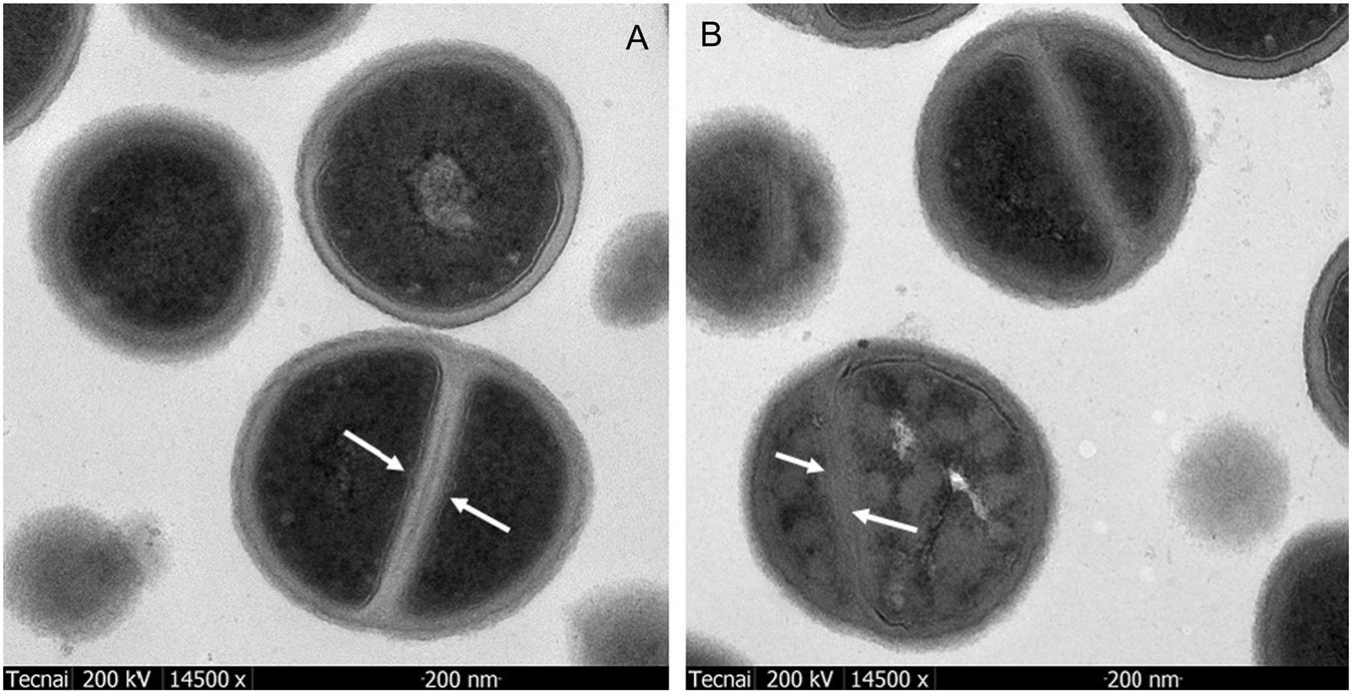
TEM images of MRSA after 30 min of aspermerodione (**1**) treatment. (**A**) Untreated MRSA. The arrows indicate intact septa. (**B**) MRSA treated with the MIC of aspermerodione (32 *μ*g/mL). The arrows indicate irregular membrane damage and disruption.

In order to visualize the morphology of the bacteria after treated by the inhibitor, the transmission electron microscopy (TEM) was employed. The evidence of TEM images (Fig. 11) of MRSA treated by compound **1** confirmed an obvious change in the ultrastructure of the strains. In the control strains, intact septa were evident, while the strains treated with MIC of compound **1**, appearing irregular membrane damage and disruption (Fig. 11).

In summary, aspermerodione (**1**), a complex polycyclic meroterpenoid containing a rare 2,6-dioxabicyclo[2.2.1]heptane motif and a new biosynthetically related compound, andiconin C (**2**) were isolated and identified from the liquid culture of the endophytic fungus *Aspergillus* sp.TJ23. The structures of both compounds were elucidated by the combined implementation of X-ray crystallography and ECD calculations. A plausible biosynthetic pathway of **1**, involving its bio-metabolite **2**, has been proposed. Our research demonstrates that aspermerodione (**1**) has anti-MRSA activity and based on the observation of TEM, the anti-MRSA activity of **1** might be tightly related to its effect of damaging bacterial cell wall and/or membrane. Moreover, aspermerodione (**1**) acts as a potential inhibitor of PBP2a that works synergistically with the *β*-lactam antibiotics oxacillin and piperacillin on antibacterial activities against MRSA. Since a promising strategy for combatting MRSA is to extend the lifespan and efficacy of our currently employed drugs using combination therapy, we believe that the disclosed novel chemical structure and bioactivity of **1** may provide a promising precursor for the development of a combination treatment regime against MRSA.

## Methods

### General

Optical rotations were determined using a Perkin-Elmer 341 polarimeter (PerkinElmer, Waltham, MA, USA). UV and IR spectra were obtained using a Varian Cary 50 (Varian, Salt Lake City, UT, USA) and a Bruker Vertex 70 instrument (Bruker, Karlsruhe, Germany), respectively. ECD spectra were recorded on a JASCO-810 spectropolarimeter. NMR spectroscopic data were recorded on a Bruker AM-400 spectrometer (Bruker, Karlsruhe, Germany), and the ^1^H and ^13^C NMR chemical shifts are expressed in *δ*, referring to the solvent impurity peaks for CDCl_3_ (*δ*_H_ 7.26 and *δ*_C_ 77.0). HRESIMS was performed in the positive ion mode on a Thermo Fisher LC-LTQ-Orbitrap XL spectrometer (Thermo Fisher, Palo Alto, CA, USA). Semi-preparative HPLC was conducted on a Dionex HPLC system equipped with an Ultimate 3000 pump (Thermo Fisher, Scientific, Germany), an Ultimate 3000 auto sampler injector, and an Ultimate 3000 diode array detector (DAD) controlled by Chromeleon software (version 6.80), using a reversed-phased C_18_ column (5 *μ*m, 10 × 250 mm, Welch Ultimate XB-C_18_). Column chromatography was performed using silica gel (80–100, 100–200, and 200–300 mesh; Qingdao Marine Chemical Inc., China), ODS (50 *μ*m, Merck, Germany), Sephadex LH-20 (Merck, Germany), and MCI gel (75–150 *μ*m, Merck, Germany). Thin-layer chromatography (TLC) was performed using silica gel 60 F254 (Yantai Chemical Industry Research Institute) and RP-C_18_ F254 plates (Merck, Germany).

### Fungus material

The fungus *Aspergillus* sp. TJ23 was isolated from the leaves of the Chinese traditional plant *Hypericum perforatum* (St John’ Wort), which was collected from the Shennongjia areas of Hubei Province, China. The sequence data for this strain has been submitted to the DDBJ/EMBL/GenBank with accession no. KY346978. The voucher sample was deposited in the culture collection center of Tongji Medical College, Huazhong University of Science and Technology.

### Extraction and isolation

Mycelia of the fungus growing on malt extract agar were inoculated in a 250 mL Erlenmeyer flask containing 100 mL of malt extract broth medium [malt extract 2.0% (w/v), glucose 2.0% (w/v) and bacterial peptone 0.1% (w/v)] and cultured statically at room temperature for 30 days. Twenty litres in total of the whole culture broth were then filtered through a filter paper. The mycelia (1.5 kg of wet weight) were extracted with EtOAc (5 × 2 L). After evaporation of the solvent, a dark brown solid (80.5 g) was obtained, which was extracted with ethyl acetate (5 × 2 L). The ethyl acetate extract was evaporated under a reduced pressure, leaving a brown viscous residue (6.2 g), which was chromatographed using RP-C_18_ silica gel CC eluted with MeOH–H_2_O (20%, 40%, 60%, 80%, and 100%) to afford five fractions (Fr.1–Fr.5). Fr.3 (1.6 g) was further fractionated on a silica gel column eluted with CH_2_Cl_2_/MeOH (20:1–0:1) to obtain six major fractions (Fr.3.1–Fr.3.6) based on the TLC analysis. Fr.3.2 (0.6 g) was further separated via semipreparative HPLC (MeOH–H_2_O, 85%) to afford compounds **1** (20.8 mg; *t*_R_ 13.2 min; 2 mL/min) and **2** (14.2 mg; *t*_R_ 9.0 min; 2 mL/min), respectively.

### Physicochemical data

*Aspermerodione (***1***)*, colorless crystals (CHCl_3_–MeOH); mp 214–216 °C; [*α*] –342 (*c* 0.19, CH_2_Cl_2_); UV (CH_2_Cl_2_) *λ*_max_ (log *ε*) = 230 (4.11), 294 (2.96) nm; IR *ν*_max_ = 2969, 2940, 1705, 1671, 1622, 1438, 1379, and 1267 cm^−1^; CD (CH_2_Cl_2_) *λ*_max_ (Δ*ε*) 232 (+37.5) and 297 (−42.5) nm; ^1^H NMR (400 MHz) and ^13^C NMR (100 MHz) data see Table 1; HRESIMS [M + Na]^+^ *m/z* 463.2049 and 441.2251 [M + H]^+^ (calcd for C_26_H_32_O_6_Na, 463.2097 and C_26_H_33_O_6_, 441.2277).

*Andiconin C (****2****)*, colorless crystals (CHCl_3_–MeOH); mp 279–281 °C; [*α*] −298.7 (*c* 0.23, MeOH); UV (MeOH) *λ*_max_ (log *ε*) = 243 (3.19) and 314 (2.39) nm; IR *ν*_max_ = 2935, 2876, 1765, 1717, 1664, 1471, 1452 and 1014 cm^−1^; CD (MeOH) *λ*_max_ (Δ*ε*) 246 (+32.4) and 311 (−35.3) nm; ^1^H NMR (400 MHz) and ^13^C NMR (100 MHz) data see Table 1; HRESIMS [M + Na]^+^ *m/z* 433.1961 and [M + H]^+^ 411.2146 (calcd for C_25_H_32_O_6_Na, 433.1991 and C_25_H_33_O_6_, 411.2171).

### Crystal data for 1

C_26_H_32_O_6_, *M* = 440.52, *a* = 11.96170(10) Å, *b* = 14.75700(10) Å, *c* = 12.60300(10) Å, α = 90°, *β* = 97.1400(10)°, *γ* = 90°, *V* = 2207.42(3) Å^3^, *T* = 100(10) K, space group *P*2_1_, *Z* = 2, *μ*(CuKα) = 0.758 mm^−1^, 22895 reflections collected, 8337 independent reflections (*R*_*int*_ = 0.0207). The final *R*_1_ values were 0.0282 (*I* ≥ 2*σ*(*I*)). The final *wR*(*F*^2^) values were 0.0726 (*I* ≥ 2*σ*(*I*)). The final *R*_1_ values were 0.0284 (all data). The final *wR*(*F*^2^) values were 0.0729 (all data). The goodness of fit on *F*^2^ was 1.049. Flack parameter = −0.04(4). Hooft parameter = 0.00(3).

### Crystal data for 2

C_25_H_30_O_5_, *M* = 410.49, *a* = 9.22259(8) Å, *b* = 14.56007(14) Å, *c* = 15.09953(13) Å, α = 90°, *β* = 90°, *γ* = 90°, *V* = 2027.59(3) Å^3^, *T* = 100(10) K, space group *P*2_1_2_1_2_1_, *Z* = 4, *μ*(CuKα) = 0.747 mm^−1^, 14739 reflections collected, 4019 independent reflections (*R*_*int*_ = 0.0251). The final *R*_1_ values were 0.0307 (*I* ≥ 2*σ*(*I*)). The final *wR*(*F*^2^) values were 0.0797 (*I* ≥ 2*σ*(*I*)). The final *R*_*1*_ values were 0.0310 (all data). The final *wR*(*F*^2^) values were 0.0799 (all data). The goodness of fit on *F*^2^ was 1.068. Flack parameter = −0.03(5). Hooft parameter = −0.00(5).

The crystallographic data for compounds **1** and **2** were collected at 100 K on a Rigaku Oxford Diffraction Supernova Dual Source, Cu at Zero equipped with an AtlasS2 CCD using Cu Kα radiation and were deposited in the Cambridge Crystallographic Data Centre. Copies of the data can be obtained free of charge from the Cambridge Crystallographic Data Centre, 12 Union Road, Cambridge CB21EZ, UK (fax: +44-1223-336-033; or e-mail: deposit@ccdc.cam.ac.uk). The structures were solved by direct methods using Olex2 software^20^, and the non-hydrogen atoms were located from the trial structure and then refined anisotropically with SHELXL-2014^21^ using a full-matrix least squares procedure based on *F*^2^. The weighted *R* factor, *wR* and goodness-of-fit *S* values were obtained based on *F*^2^. The hydrogen atom positions were fixed geometrically at the calculated distances and allowed to ride on their parent atoms.

### ECD calculation details

The conformations of **1** and **2** generated by BALLOON^22,23^ were subjected to semiempirical PM3 quantum mechanical geometry optimizations using the Gaussian 09 program^24^. Duplicate conformations were identified and removed when the root-mean-square (RMS) distance was less than 0.5 Å for any two geometrically optimized conformations. The remaining conformations were further optimized at the B3LYP/6-31 G(d) level in MeOH with the IEFPCM solvation model using Gaussian 09, and the duplicate conformations emerging after these calculations were removed according to the same RMS criteria above. The harmonic vibrational frequencies were calculated to confirm the stability of the final conformers. The electronic circular dichroism (ECD) spectrum was calculated for each conformer using the TDDFT methodology at the LC-wPBE/6-311++G(d,p)//B3LYP/6-31 G(d) level with MeOH as the solvent via the IEFPCM solvation model implemented in the Gaussian 09 program. The ECD spectrum for each conformer was simulated using a Gaussian function with a bandwidth, σ, of 0.4 eV. The spectra were combined after Boltzmann weighting according to their population contributions, and a UV correction was applied^25^.

## Biological Activities Evaluation

### Strains, media, and antibiotics

The tested MRSA strain was obtained from the ATCC: MRSA (ATCC 43300). The reference compounds for the tests were recommended by the National Committee for Clinical Laboratory Standards^26^. Oxacillin (Sigma, cat # 1481000); Piperacillin (Sigma, cat # 1541500); Chloramphenicol (Sigma, cat # 1107300); Vancomycin (Sigma, cat # 861987). Meropenem (Sigma, cat # M2574). Investigated compounds **1** and **2** were ≥95% pure (HPLC, wavelength = 210 nm). All compounds were dissolved in DMSO as 20 mg/mL stock solutions.

### Determination of the minimum inhibitory concentrations (MIC)

Determination of the MICs were conducted according to our previously reported broth microdilution method^12,13^. In brief, the inoculum was standardized to approximately 5 × 10^5^ CFU/mL. The plates were incubated at 37 °C for 16 h, and the MIC values were recorded as the lowest concentration of antibiotic at which no visible bacterial growths were observed. Each experiment was performed three times.

### Combination susceptibility test against MRSA

Determination of the combination susceptibility was conducted according to our previously reported broth microdilution checkerboard assay^12,13^. MHB was inoculated with MRSA (5 × 10^5^ CFU/mL), and 100 *μ*L was distributed into each well of a 96-well plate except in well 1 A. Inoculated MHB (200 *μ*L) containing test compound (at 2 × MIC) were added to well 1 A, and 100 *μ*L of the same sample was placed in each of well for 2A-12A. Column A wells were mixed 6 to 8 times, and then 100 *μ*L was withdrawn and transferred to column B. Column B wells were mixed 6 to 8 times, followed by a 100 *μ*L transfer to column C. This procedure was repeated to serially dilute the rest of the columns of the plate up to column G (column H was not mixed to allow the MIC of the antibiotic alone to be determined). Inoculated media (100 *μ*L) containing the antibiotic at two times, the MIC was placed in wells A1-H1 (row 1) and serially diluted in the same manner as row 11. The plates were incubated for 16 h at 37 °C. The MIC values of both compounds and the antibiotic in the combination were recorded as well as the MIC values of the compound alone (row 12) and antibiotic alone (column H). The ∑FIC values were calculated as follows: ∑FIC = FIC_Compd_ + FIC_Antibiotic_, where FIC_Compd_ is the MIC of the compound in the combination/MIC of the compound alone, and FIC_Antibiotic_ is the MIC of the antibiotic in the combination/MIC of the antibiotic. The combination is considered synergistic when the ∑FIC value is ≤0.5, accumulative or indifferent when the ∑FIC value is >0.5 but <2, and antagonistic when the ∑FIC is ≥2^27^.

### Molecular docking and virtual screening

The compounds were saved as mol2 files and used as an input for docking. The docking was performed using the Surflx-Dock module of the Sybyl softare^28,29^. Ligand binding pocket residues were selected using graphical tools in the Sybyl software to create the boundaries of the docking search. All of the hydrogen atoms were added to define the correct ionization and tautomeric states, and the carboxylate, phosphonate and sulphonate groups were considered in their charged form. In the docking calculation, the default FlexX scoring function was used for exhaustive searching, solid body optimizing and interaction scoring^28,29^. The pose with the lowest-energy and the most favorable score was remained.

### Protein expression and purification

Wild-type PBP2a (residues 23-668) and four other PBPs (PBPs 1–4) were cloned into a pET28a vector (EMD Biosciences, Novagen, United States) from the *mec*A sequence of *Staphylococcus aureus* ATCC 43300. After confirmation by DNA sequencing, the recombined plasmid was transferred into *Escherichia coli* strain, BL21 (DE3). The transformed cells were grown in LB medium at 37 °C to an OD_600_ (0.8–1.0) and induced with 0.4 mM of isopropyl-D-thiogalactopyranoside (IPTG) at 293 K for 16 h. After they were harvested via centrifugation, the cells were re-suspended on ice in a lysis buffer containing 20 mM Tris (pH 7.5), 200 mM NaCl, and 10 mM imidazole, followed by disruption on a French press. Cell debris was removed via centrifugation at 21,000 rpm for 30 min. The protein was bound to Ni-agarose affinity resin, washed with a buffer containing 20 mM Tris (pH 7.5), 200 mM NaCl, and 10 mM imidazole, and eluted with a buffer containing 20 mM Tris (pH 7.5), 250 mM NaCl, and 150 mM imidazole. Fractions containing PBP2a were pooled and further purified on Sephacryl S-200 size-exclusion resin in 2 mM of Tris (pH 7.5) and 200 mM of NaCl.

### Microscale thermophoresis

Recombinant PBP2a and four other PBPs (PBPs 1–4) were labelled with the Monolith NT^TM^ Protein Labelling Kit, RED (Cat # L001), according to the supplied labelling protocol. Labelled PBPs was used at a concentration of 50 nM. Samples were diluted in a 20 mM HEPES (pH 7.4) and 0.05 (v/v)% Tween-20. We used 5 mM of compounds **1** and **2** as the highest concentration for the serial dilution. Oxacillin and Meropenem were used as positive controls. After 10 min of incubation at room temperature, the samples were loaded into Monolith^TM^ standard-treated capillaries, and the thermophoresis was measured at 25 °C after 30 min of incubation on a Monolith NT.115 instrument (NanoTemper Technologies, München, Germany). The laser power was set to 20% or 40% using 30 seconds on-time. The LED power was set to 100%. The dissociation constrant, Kd, values were fitted by using the NTAnalysis software (NanoTemper Technologies, München, Germany)^30^.

### Transmission electron microscopy (TEM)

Mid-logarithmic grown MRSA was collected by centrifugation at 10,000 g for 10 min, and fixed with modified Karnovsky’s fixative. Then the specimens were examined using an energy-filtering transmission electron microscope (Tecnai G2 20 TWIN, FEI, Oregon, USA) operated at 200 kV accelerating voltage.

### Statistical analysis

The Graph Pad Prism 5.0 software was employed to analyze the statistical data, with the outcome expressed as the means ± SD. Values were analysed using SPSS version 12.0 software by one-way analysis of variance (ANOVA), and a p < 0.05 was considered statistically significant.

## Electronic supplementary material

Supplementary Information
