# Supplementary material for: Aspermerodione, a novel fungal metabolite with an unusual 2,6-dioxabicyclo[2.2.1]heptane skeleton, as an inhibitor of penicillin-binding protein 2a
Source: Sci Rep. 2018 Apr 3;8:5454. doi: 10.1038/s41598-018-23817-1 (PMC5882964; doi:10.1038/s41598-018-23817-1)

Aspermerodione, a novel fungal metabolite with an unusual 2,6-dioxabicyclo[2.2.1]heptane skeleton, as an inhibitor of penicillin-binding protein 2a

Yuben Qiao,[1,†] Xiaotian Zhang,[1,2,†] Yan He,[1,3,†] Weiguang Sun,[1,†] Wenya Feng,[1] Junjun Liu,[1] Zhengxi Hu,[1] Qianqian Xu,[1] Hucheng Zhu,[1] Jinwen Zhang,[2] Zengwei Luo,[1] Jianping Wang,[1,*] Yongbo Xue,[1,*] and Yonghui Zhang[1,*]

[1]Hubei Key Laboratory of Natural Medicinal Chemistry and Resource Evaluation, School of Pharmacy, Tongji Medical College, Huazhong University of Science and Technology, Wuhan 430030, Hubei Province, People's Republic of China

[2]Biological Engineering, Wuchang Shouyi University, Wuhan 430064, China

[3]Tongji Hospital Affiliated to Tongji Medical College, Huazhong University of Science and Technology, Wuhan 430030, People's Republic of China

Content

Fig. S1. ^{1}H NMR Spectrum of **1** in $CDCl_3$ 3

Fig. S2. ^{13}C NMR Spectrum of **1** in $CDCl_3$ 3

Fig. S3. HSQC Spectrum of **1** in $CDCl_3$ 4

Fig. S4. HMBC Spectrum of **1** in $CDCl_3$ 4

Fig. S5. ^{1}H–^{1}H COSY Spectrum of **1** in $CDCl_3$ 5

Fig. S6. NOESY Spectrum of **1** in $CDCl_3$ 5

Fig. S7. (+)-HR-ESI-MS Spectrum of **1** 6

Fig. S8. IR Spectrum of **1** 6

Fig. S9. UV Spectrum of **1** 7

Fig. S10. ^{1}H NMR Spectrum of **2** in $CDCl_3$ 7

Fig. S11. ^{13}C NMR Spectrum of **2** in $CDCl_3$ 8

Fig. S12. HSQC Spectrum of **2** in $CDCl_3$ 8

Fig. S13. HMBC Spectrum of **2** in $CDCl_3$ 9

Fig. S14. ^{1}H–^{1}H COSY Spectrum of **2** in $CDCl_3$ 9

Fig. S15. NOESY Spectrum of **2** in $CDCl_3$ 10

Fig. S16. (+)-HR-ESI-MS Spectrum of **2** 10

Fig. S17. IR Spectrum of **2** 11

Fig. S18. UV Spectrum of **2** 11

Fig. S19. Detailed HMBC and ^{1}H–^{1}H COSY correlations of compounds **1** and **2**. 12

Fig. S1. ^{1}H NMR Spectrum of **1** in $CDCl_3$

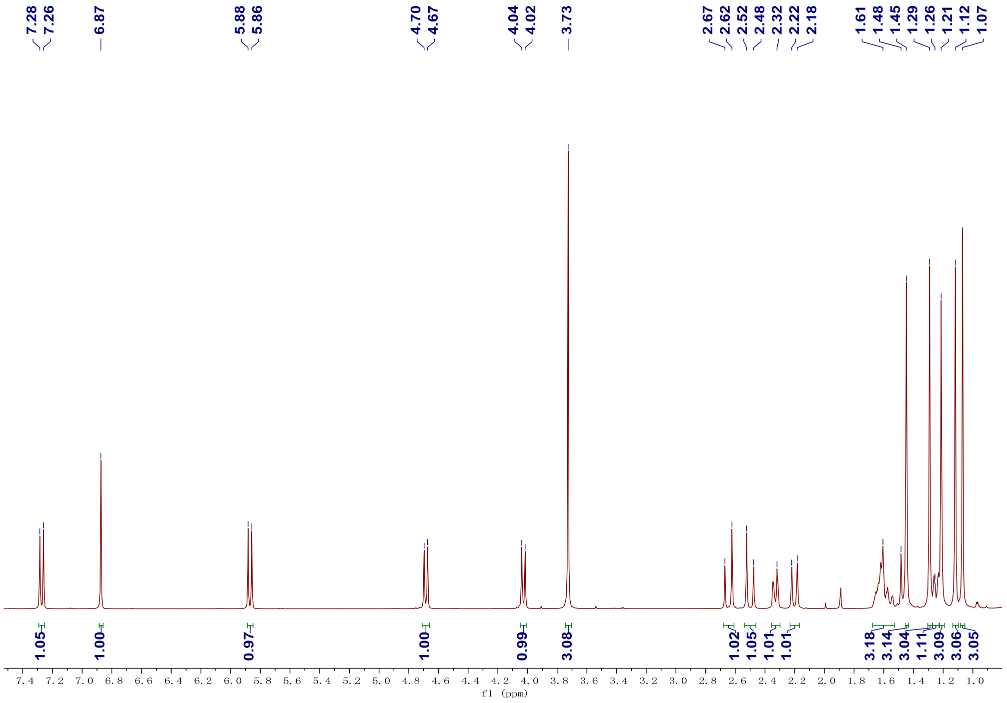

Fig. S2. ^{13}C NMR Spectrum of **1** in $CDCl_3$

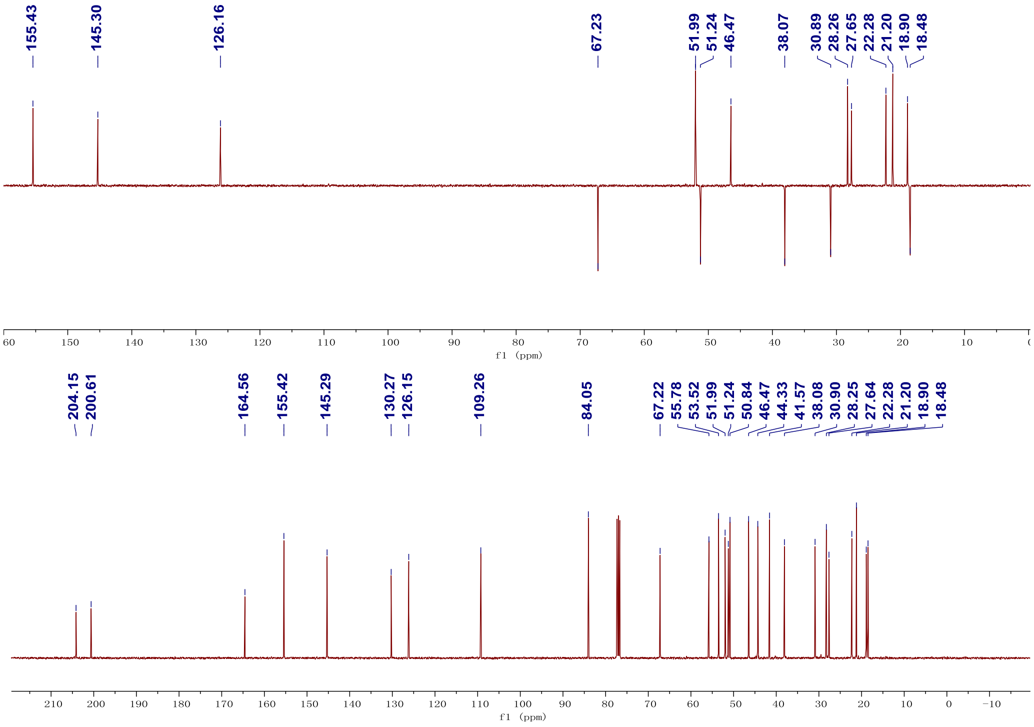

Fig. S3. HSQC Spectrum of **1** in $CDCl_3$

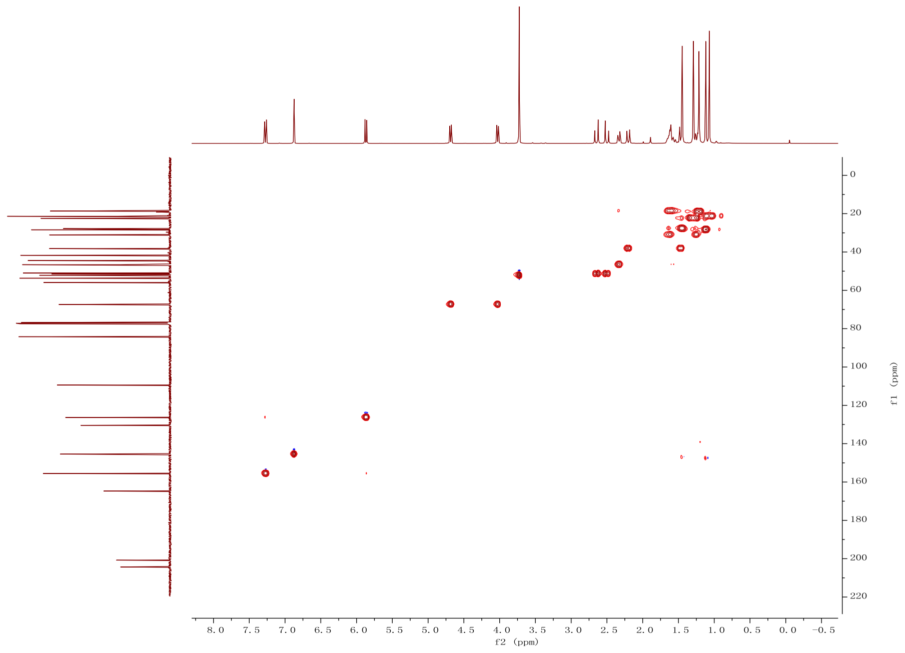

Fig. S4. HMBC Spectrum of **1** in $CDCl_3$

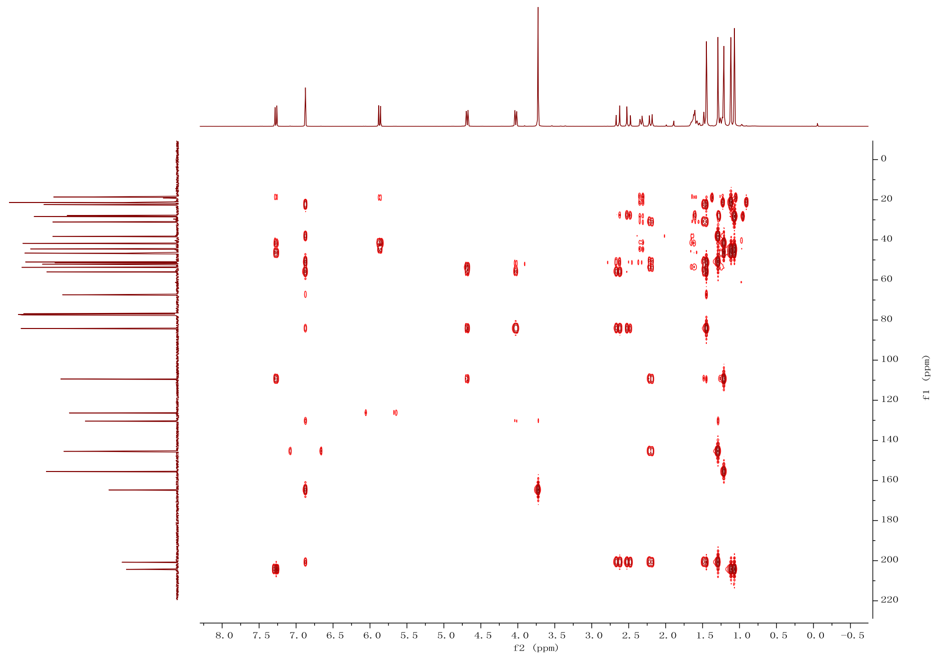

Fig. S5. ^1H–^1H COSY Spectrum of **1** in $CDCl_3$

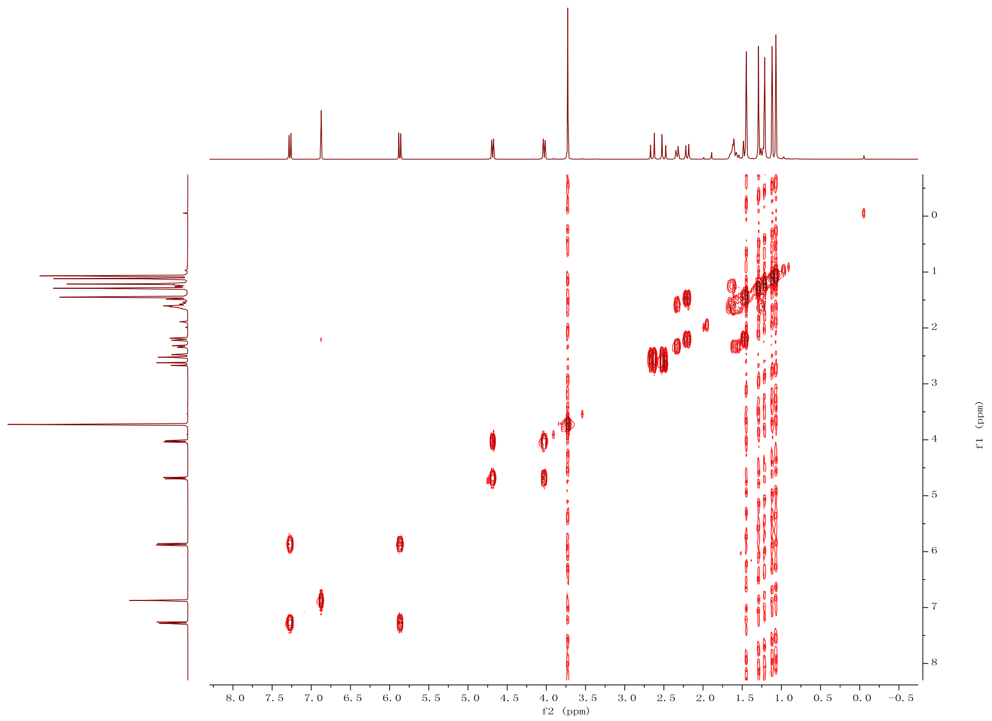

Fig. S6. NOESY Spectrum of **1** in $CDCl_3$

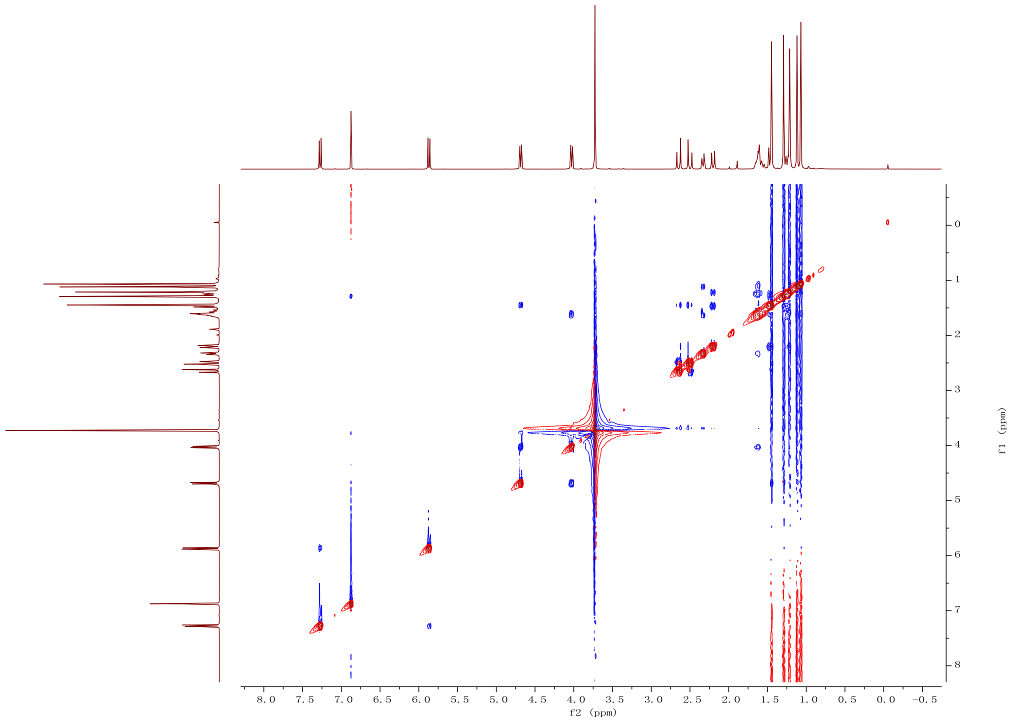

Fig. S7. (+)-HR-ESI-MS Spectrum of **1**

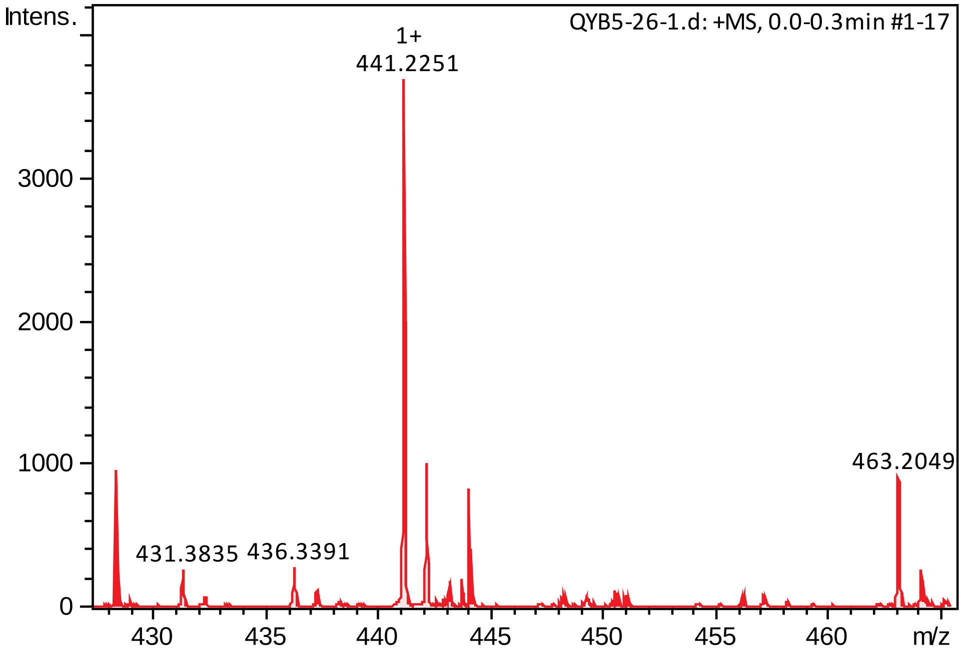

Fig. S8. IR Spectrum of **1**

E:\20170302\QYB\5-26-1.0 15:09:37 2017-3-2

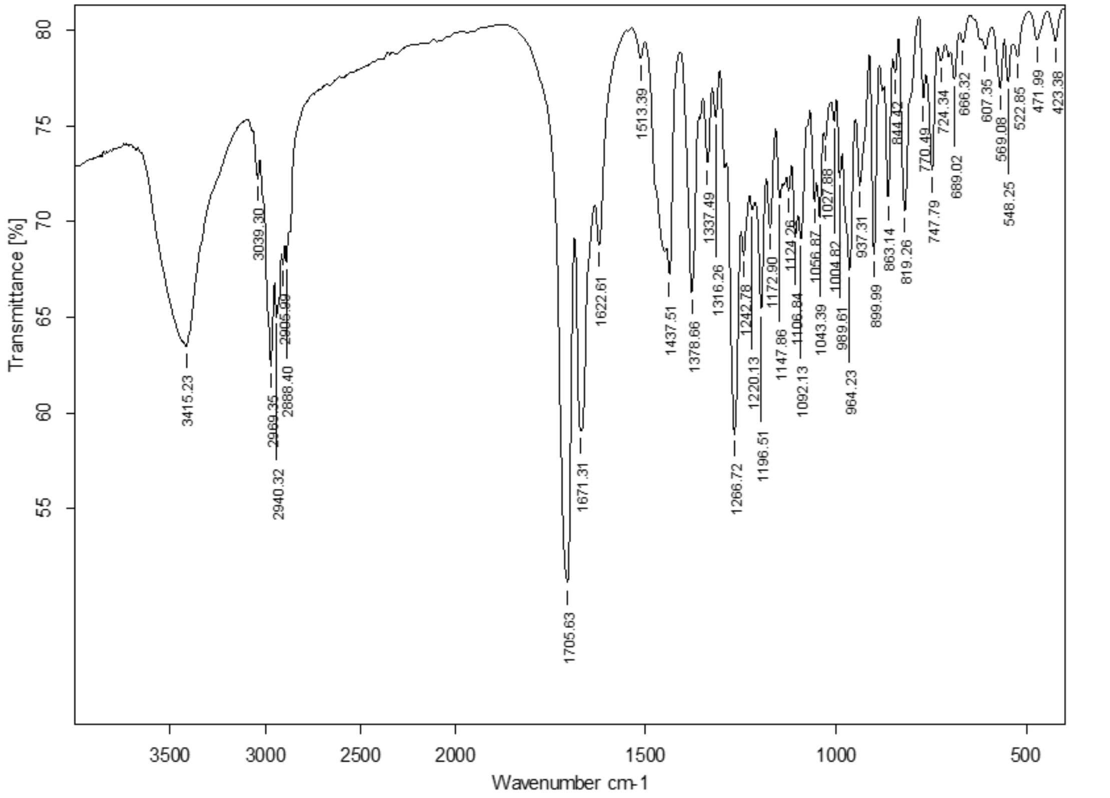

Fig. S9. UV Spectrum of **1**

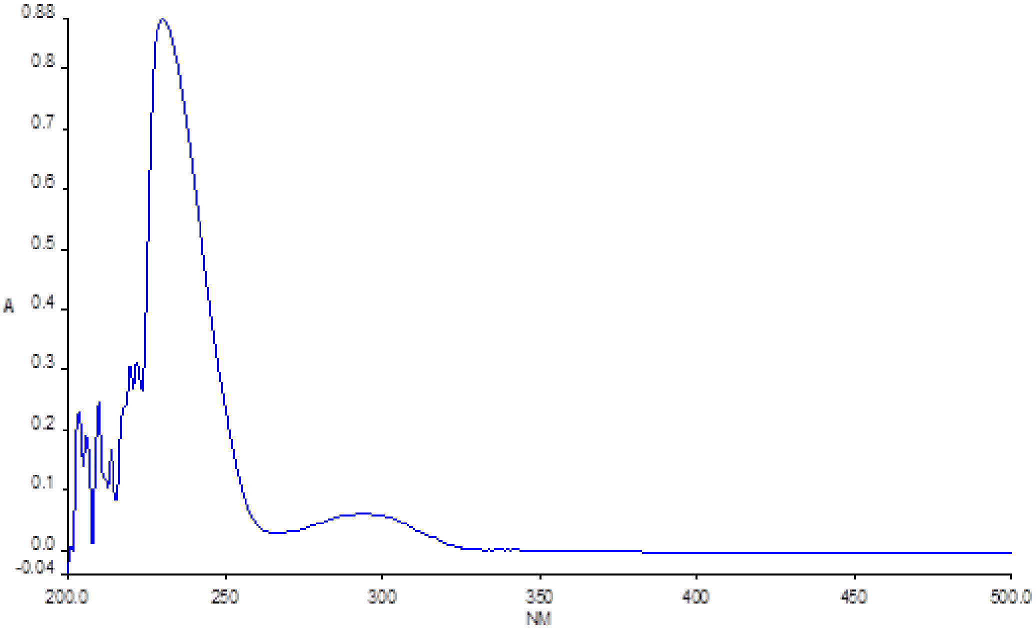

Fig. S10. ^{1}H NMR Spectrum of **2** in $CDCl_3$

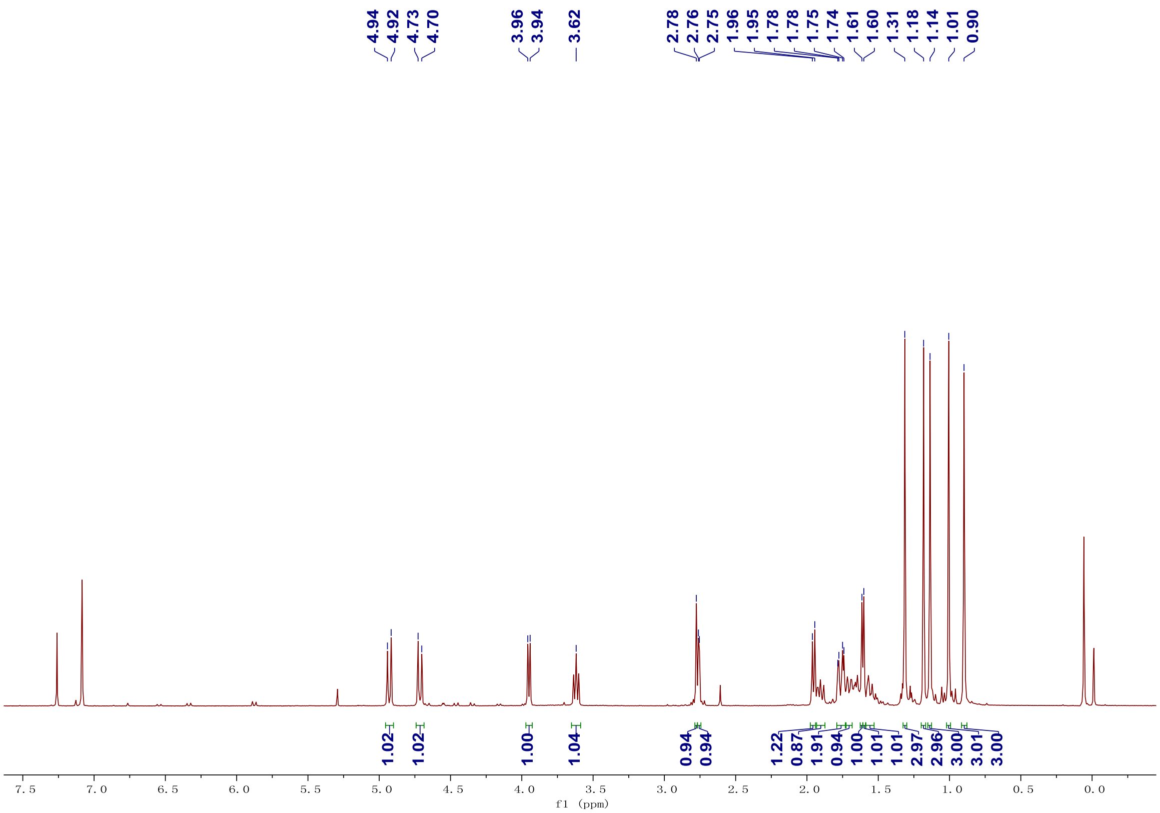

Fig. S11. ^{13}C NMR Spectrum of **2** in $CDCl_3$

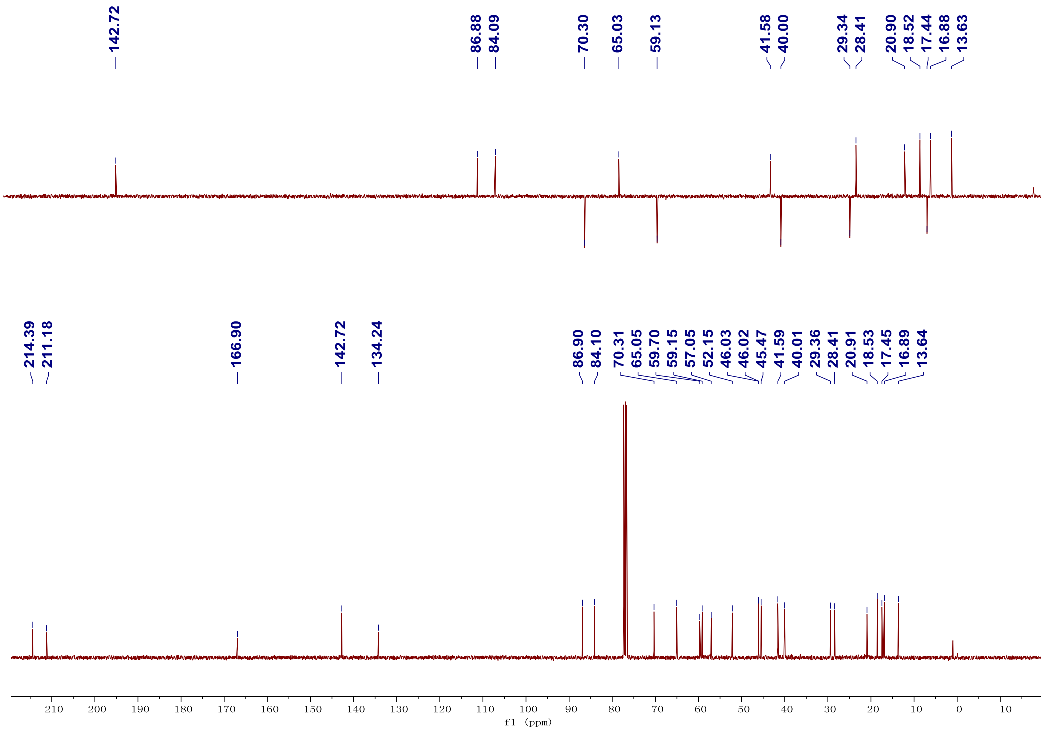

Fig. S12. HSQC Spectrum of **2** in $CDCl_3$

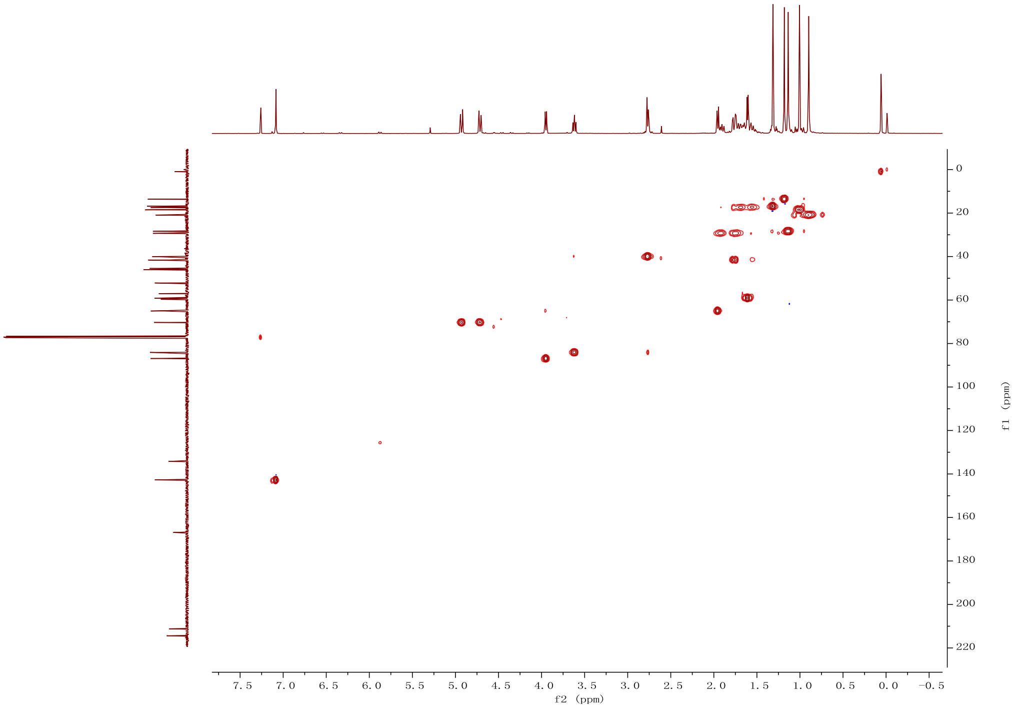

Fig. S13. HMBC Spectrum of **2** in $CDCl_3$

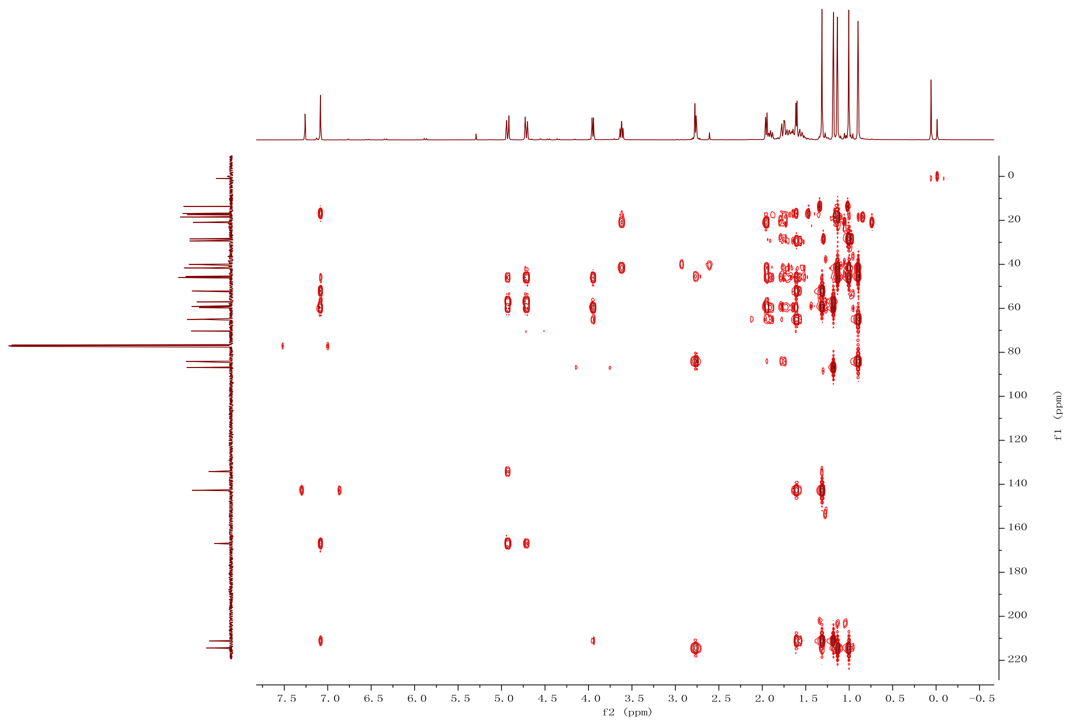

Fig. S14. ^{1}H–^{1}H COSY Spectrum of **2** in $CDCl_3$

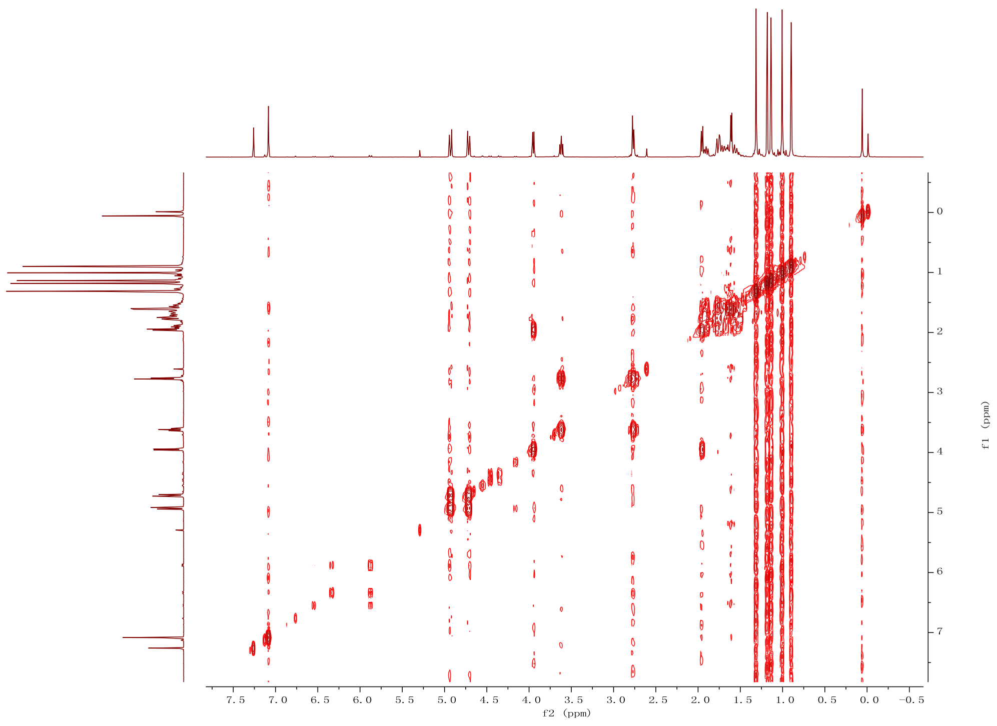

Fig. S15. NOESY Spectrum of **2** in $CDCl_3$

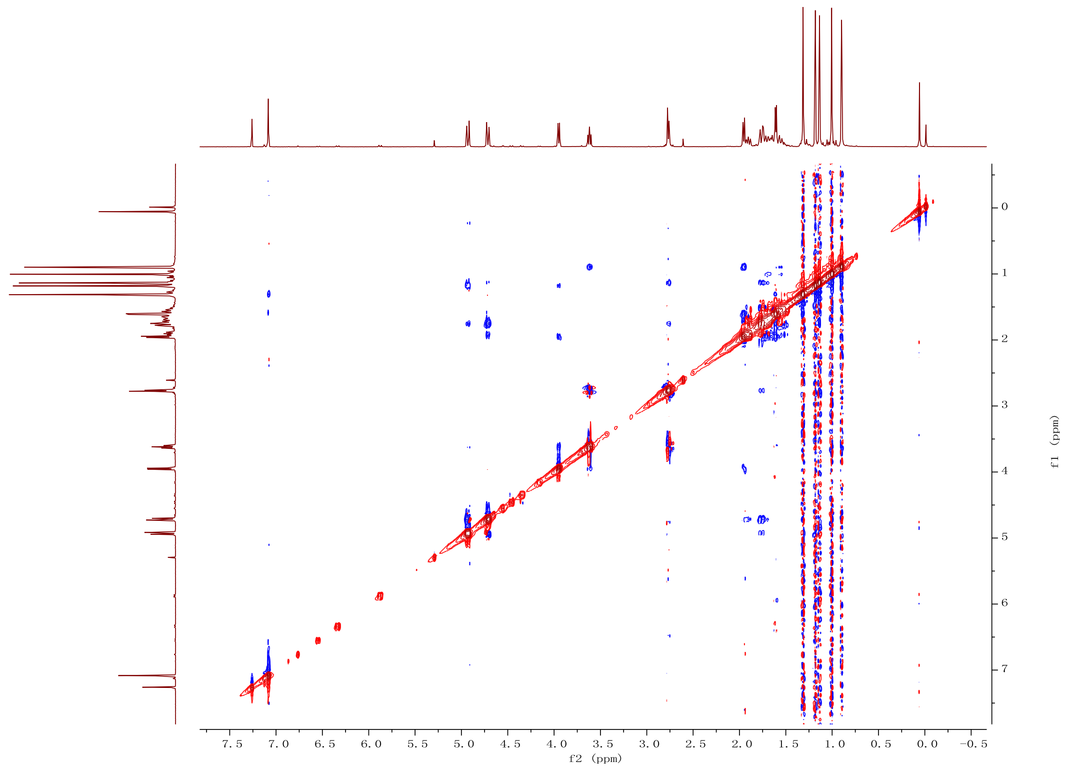

Fig. S16. (+)-HR-ESI-MS Spectrum of **2**

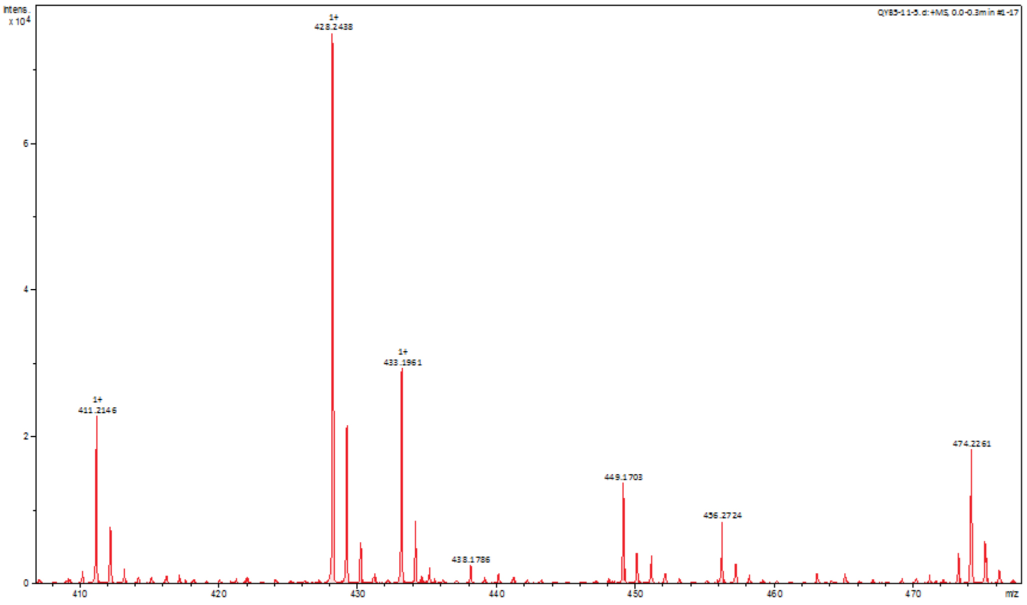

Fig. S17. IR Spectrum of **2**

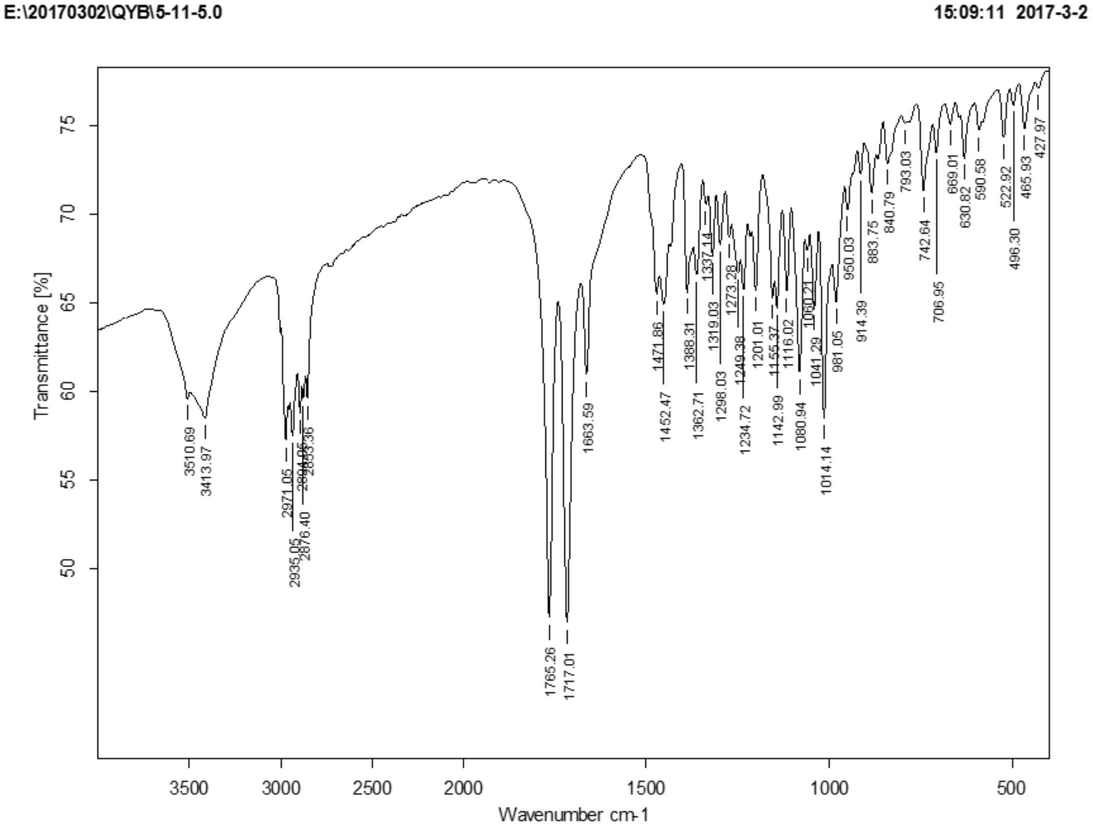

Fig. S18. UV Spectrum of **2**

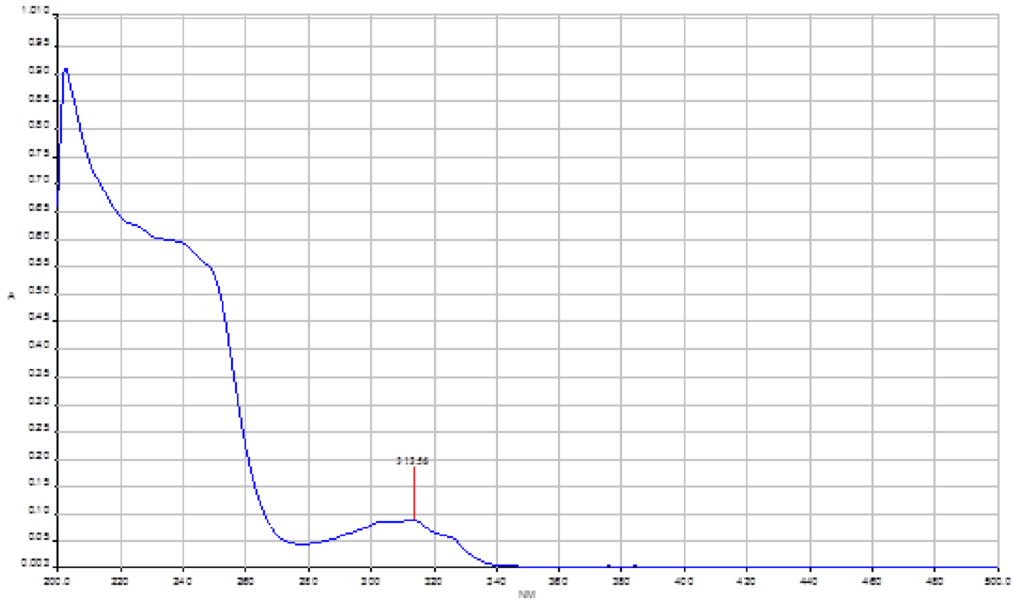

Fig. S19. Detailed HMBC and ^{1}H–^{1}H COSY correlations of compounds **1** and **2**.

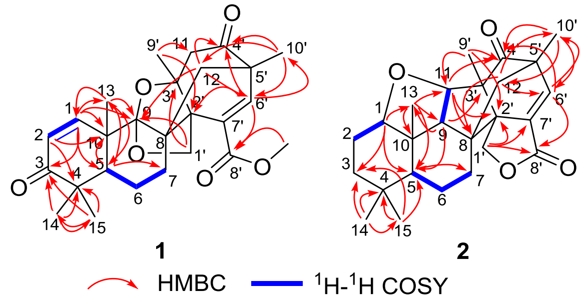

Supplement: Supplementary file 1 — Supplementary Information [file 41598_2018_23817_MOESM1_ESM.pdf]
